# A large shRNA library approach identifies lncRNA *Ntep* as an essential regulator of cell proliferation

**DOI:** 10.1038/cdd.2017.158

**Published:** 2017-11-03

**Authors:** Julia Beermann, Dominique Kirste, Katharina Iwanov, Dongchao Lu, Felix Kleemiß, Regalla Kumarswamy, Katharina Schimmel, Christian Bär, Thomas Thum

**Affiliations:** 1 Institute of Molecular and Translational Therapeutic Strategies (IMTTS), Hannover Medical School, Hannover, Germany; 2 National Heart and Lung Institute, Imperial College London, London, UK; 3 REBIRTH Excellence Cluster, Hannover Medical School, Hannover, Germany

## Abstract

The mammalian cell cycle is a complex and tightly controlled event. Myriads of different control mechanisms are involved in its regulation. Long non-coding RNAs (lncRNA) have emerged as important regulators of many cellular processes including cellular proliferation. However, a more global and unbiased approach to identify lncRNAs with importance for cell proliferation is missing. Here, we present a lentiviral shRNA library-based approach for functional lncRNA profiling. We validated our library approach in NIH3T3 (3T3) fibroblasts by identifying lncRNAs critically involved in cell proliferation. Using stringent selection criteria we identified lncRNA NR_015491.1 out of 3842 different RNA targets represented in our library. We termed this transcript *Ntep* (non-coding transcript essential for proliferation), as a *bona fide* lncRNA essential for cell cycle progression. Inhibition of *Ntep* in 3T3 and primary fibroblasts prevented normal cell growth and expression of key fibroblast markers. Mechanistically, we discovered that *Ntep* is important to activate P53 concomitant with increased apoptosis and cell cycle blockade in late G2/M. Our findings suggest *Ntep* to serve as an important regulator of fibroblast proliferation and function. In summary, our study demonstrates the applicability of an innovative shRNA library approach to identify long non-coding RNA functions in a massive parallel approach.

Only a minimal portion of mammalian genes are transcribed into proteins,^1, 2^ while the majority of transcripts are non-coding RNAs. Many fulfil regulatory functions without being further processed into proteins.^3^ Long non-coding RNAs (lncRNAs) represent a diverse sub-population of non-coding RNAs, classified as transcripts longer than 200 nucleotides. Several lncRNAs were shown to be involved in different cellular mechanisms.^4, 5^ This includes, for instance, transcriptional regulation ^6^ and formation of scaffolds for molecular interaction partners.^7^

The cell cycle is a tightly regulated process; thus, misregulation of cell cycle checkpoints can lead to cancer^8^ or fibrotic diseases.^9, 10^ Accordingly, a number of lncRNAs are critically involved in cell cycle regulation.^11^ For instance, the lncRNA *MALAT1* modulates the expression of cell cycle genes and controls the progression of G2 to M phase,^12^ whereas the lncRNA *PANDA* suppresses DNA-damaged induced apoptosis.^13^ LncRNA *PINT* connects P53 activation with PRC2 (polycomb repressive complex 2) silencing to promote cell proliferation and survival by regulating the TGF*β*, MAPK and P53 signalling pathways.^14^ By contrast, *lincRNA-p21* was shown to act as a repressor of P53-driven gene expression.^15^

Despite these few examples, unbiased approaches for high-throughput functional lncRNA screening to find novel lncRNAs regulating fibroblast cell cycle and proliferation are scarce. In 2014, a novel lncRNA important for pluripotency and neural differentiation of mouse embryonic stem cells was discovered by using an shRNA library targeting 1280 lincRNAs in parallel.^16^

In our study, we aimed to further develop this method by increasing the target size to 3842 including lncRNAs, controls and ultraconserved elements (UCE), which were shown to give rise to lncRNAs and to be regulated during disease.^17^ We designed a 26k shRNA library and screened for non-coding targets involved in fibroblast proliferation. Using stringent selection criteria, we identified NR_015491.1 to be essential for fibroblast proliferation. We named this lncRNA *Ntep* (non-coding transcript essential for proliferation)*. Ntep* expression is essential for maintenance of basic fibroblast parameters such as migration, colony formation and expression of extracellular matrix components. *Ntep* inhibition leads to an upregulation of DNA-damage-related pathways concomitant with impaired cell cycle progression and increased rates of apoptosis.

Collectively, we demonstrated the successful application of a broad shRNA-mediated knockdown to screen for novel cellular functions of lncRNAs. Thus, we provide an unbiased high-throughput tool to investigate massive amounts of lncRNA targets in parallel.

## Results

### Development of a 26k shRNA library for functional studies of ~3800 murine lncRNAs

A 26 391 element shRNA library was manufactured to target 3842 murine lncRNAs and UCEs listed in RefSeq in 2013 (Cellecta) (see Supplementary File 1). The shRNA sequences were assembled into a pRSI16 lentiviral vector backbone, containing an RFP reporter and a puromycin resistance marker, to allow for sorting and/or calculation of transduction efficiency and for antibiotic selection of transduced cells (Supplementary Figure S1). Each shRNA was barcoded for unequivocal identification by HT sequencing. The library contains six to seven shRNAs per individual lncRNA, thus decreasing false-positive hits in genome-wide screens due to off-target effects. Additionally, the library contains 38 shRNA to target luciferase as an internal control. Since those shRNAs do not have target sequences in murine cells, their frequency distribution was used as an shRNA enrichment threshold in our screening approaches.

### Application of the shRNA library to identify lncRNAs involved in cellular proliferation

**Figure 1:**
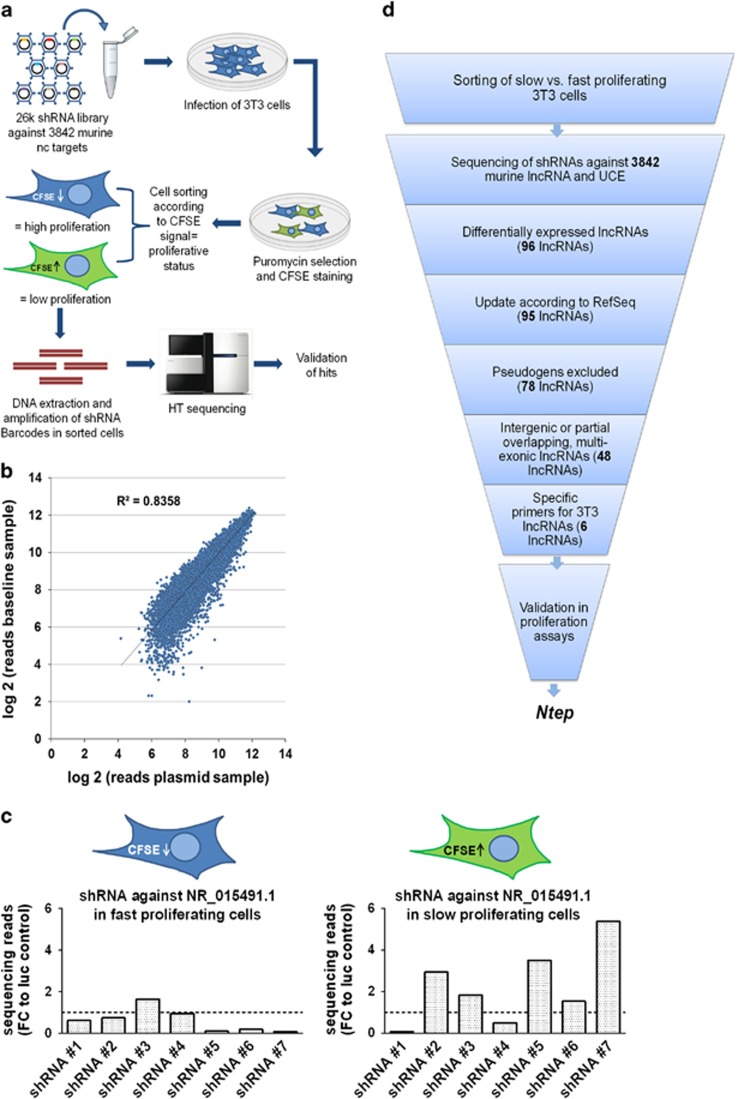
Experimental design and selection strategy for the identification of lncRNAs essential for proliferation. (**a**) Schematic workflow of proliferation-based lncRNA shRNA library screen in 3T3 cells. (**b**) Library infection quality control: Scatter plot of the log 2-transformed DNA sequencing read values from baseline sample (5 days post library infection) normalized to 20 million reads *versus* the sequencing reads of the input pooled plasmid library normalized to 20 million reads. (**c**) Sequencing reads of shRNA barcodes against lncRNA NR_015491.1 (*Ntep*) in CFSE-low and -high sub-populations. Data are fold-change (FC) relative to barcode reads of shRNAs in each sub-population against luciferase control. (**d**) Scheme of the selection strategy to narrow down initial lncRNA candidates that influence proliferation. UCE, ultraconserved elements

The shRNA library was applied to systematically screen for lncRNAs that are important for proliferation of 3T3 cells. The shRNAs were packed in lentiviral particles and transduced 3T3 cells at an MOI of 0.5 to ensure single shRNA integration. Three days after infection, cells were selected on puromycin and further grown for 2 days. Cells were then labelled with carboxyfluorescein succinimidyl ester (CFSE) and grown for an additional 5 days. Since the signal gradually declines with each cell division, the CFSE staining was used to monitor the proliferative status of cells.^18^ Combining this assay with the shRNA library approach represented the set-up to investigate the effect of >3800 annotated lncRNAs and UCEs on fibroblast proliferation. Cells were analysed by fluorescence-activated cell sorting (FACS) and gated into sub-populations with either high or low CFSE signals, corresponding to slow or fast proliferating cells, respectively (Figure 1a and Supplementary Figure S2). To control for effects of the CFSE staining of proliferation *per se*, uninfected 3T3 cells stained with CFSE were used to set the gates during sorting. DNA of the cell sub-populations was extracted and subjected to high-throughput sequencing to determine the abundance of each barcode. As an additional quality control for the screen, we sequenced the pooled shRNA plasmid library before virus production (20 million reads) and compared this to a baseline sample of the 3T3 cells 5 days after library infection. The read counts in both samples correlated well, indicative of an even shRNA distribution (Figure 1b).

LncRNAs were considered candidates when their corresponding shRNAs were underrepresented in fast proliferating cells and at least 4 out of 6–7 were overrepresented in slow proliferating cells in comparison with the abundance of luciferase shRNAs (Figure 1c and Supplementary Figure S3). This yielded 96 lncRNAs for which we assumed differential expression in both conditions. To narrow down the number of lncRNA candidates, we applied stringent selection criteria: (1) transcribed pseudogenes were excluded to avoid parental gene detection by mistake when amplifying the lncRNA transcript; (2) intergenic lncRNAs were selected (i.e. natural antisense transcripts were excluded); (3) only multiexonic lncRNAs were selected allowing to design intron-spanning primers. After this selection, six lncRNA candidates could be specifically amplified in 3T3 cells, which were selected for further analyses (Figure 1c and Supplementary Figure S3). For validation of these candidates, we did not only search for those whose inhibition would lead to a decreased proliferation but also for candidates whose endogenous expression level is lowered in antiproliferative settings. Therefore, we measured the expression level of the six candidates in 3T3 cells that were subjected to medium starvation and hypoxic conditions. Hypoxic conditions (0.1% O_2_) for different time periods expectedly reduced cell proliferation as assessed by bromodeoxyuridine (BrdU) incorporation. After hypoxia, the proliferation level dropped by 11%, 50% and 57% after 24, 48 and 72 h, respectively (Supplementary Figure S4B). Reduced proliferation rates correlated with the expression of only one lncRNA candidate (NR_015491), which was progressively downregulated over time in hypoxia compared with normoxia (Supplementary Figure S4A). Similarly, 3T3 cells grown for 24 h in starvation medium supplemented with both 1% or 0.1% FBS showed reduced cell proliferation of 32% or 25%, respectively (Supplementary Figure S4C). Again, this was associated with a significant endogenous downregulation of NR_015491 (Supplementary Figure S4D). Based on these results, we named lncRNA NR_015491.1 as *Ntep*, the most consistent and strongest regulator of cell proliferation emerging from both our shRNA screen and expression profiling under antiproliferative conditions (Figure 1d).

### *Ntep* is important for cell proliferation

**Figure 2:**
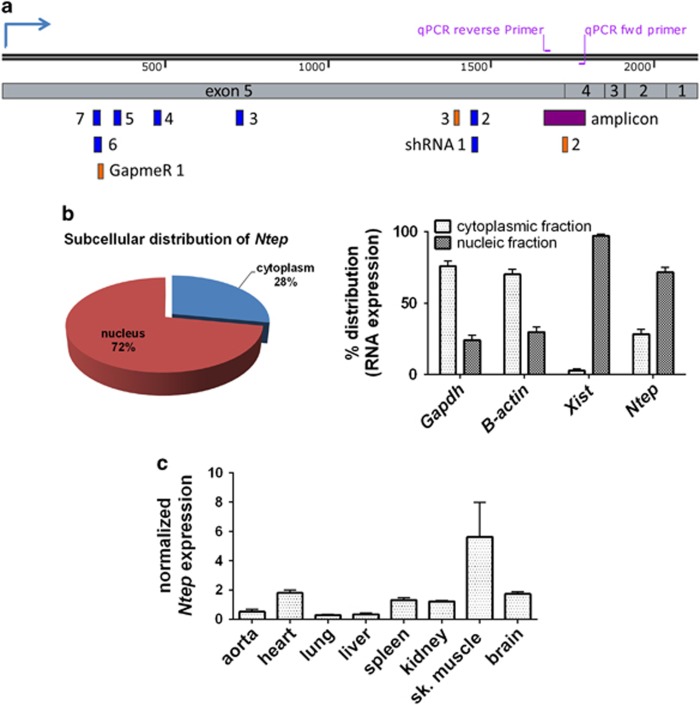
*Ntep* is a mainly nuclear lncRNA. (**a**) Graphical representation of *Ntep* showing multiple exons with different lengths according to GRCm38/mm10. Primers used for qPCR analysis are shown in purple, individual shRNAs from shRNA library are shown in blue and GapmeRs used to target *Ntep* are shown in orange. (**b**) Subcellular distribution of *Ntep* in 3T3 subfractions, 18s, *GAPDH*, *β-Actin* and *Xist* expression levels were measured as positive controls. Data are % distribution calculated to complete amount of transcript in qPCR analysis±S.E.M. (*n*=3 independent experiments). Piechart showing the subcellular distribution of *Ntep* in 3T3 cells. Data are % distribution calculated to complete amount of transcript in qPCR analysis (*n*=3 independent experiments). (**c**) Expression of *Ntep* in different organs from C57BL6J mice (*n*=3). Normalized expression level was measured with qPCR±S.E.M. *P<0.05; **P<0.01; ***P<0.001

*Ntep* is a multiexonic, intergenic lncRNA, 2131 nt in length (according to RefSeq; NR_015491.1) and is located on chromosome 16 (16: 98 062 512–98 082 439). Primers to validate the expression of *Ntep* were designed to span an intron of 2305 nt, allowing to distinguish clearly the transcript from any genomic DNA (contamination) (Figure 2a). The primers were checked for specificity in gene-specific PCR (GS-PCR), using the *Ntep* primers for reverse transcription following a conventional PCR (Supplementary Figures S5A and B). Agarose gel electrophoresis showed only one band of the expected size (125 bp) and Sanger sequencing confirmed the specific sequence (Supplementary Figure S5C).

LncRNAs may contain open reading frames (ORFs) and certain lncRNAs may encode micropeptides.^19, 20^ To rule out this possibility for *Ntep*, we calculated potential ORFs within the sequence of *Ntep* using coding potential calculator and sequence manipulation suite.^21, 22^ *Ntep* contains two ORFs encoding potential proteins of 247 and 456 amino acids. A protein BLAST search (NCBI blast tool) with the putative peptides did not identify perfect matches. We next constructed a 3T3 cell line overexpressing *Ntep* and analysed the proteome by mass spectrometry. We did not detect any of the corresponding putative peptides, neither in the overexpression cell line nor in the wild-type 3T3 (Supplementary Figure S6). We thus conclude that *Ntep* is a *bona fide* non-coding transcript. We performed a subcellular fractionation and measured *Ntep* levels in cytoplasmic and nuclear fractions. According to cytoplasmic *GAPDH* and *β-actin* mRNAs as well as nuclear localized lncRNA *Xist*, we calculated a percentage distribution of 72% and 28% in the nucleus and cytoplasm, respectively (Figure 2b). We next tested *Ntep* expression in various mouse organs and found a relatively high expression in the heart, spleen, kidney and brain compared with lung, liver and aorta, whereas the expression was even stronger in the skeletal muscle (Figure 2c and Supplementary Figure S7). In conclusion, *Ntep* predominantly resides in the nucleus and is expressed in fibroblasts and several organs.

### Inhibition of *Ntep* impairs fibroblast functions

To study the effects of *Ntep* on fibroblast proliferation, we silenced *Ntep* using GapmeRs. We tested three different GapmeRs, which all efficiently silenced *Ntep*. However, GapmeR3 resulted in the most efficient downregulation (1% compared with GapmeR control) and was used for all subsequent experiments (Supplementary Figure S8).

**Figure 3:**
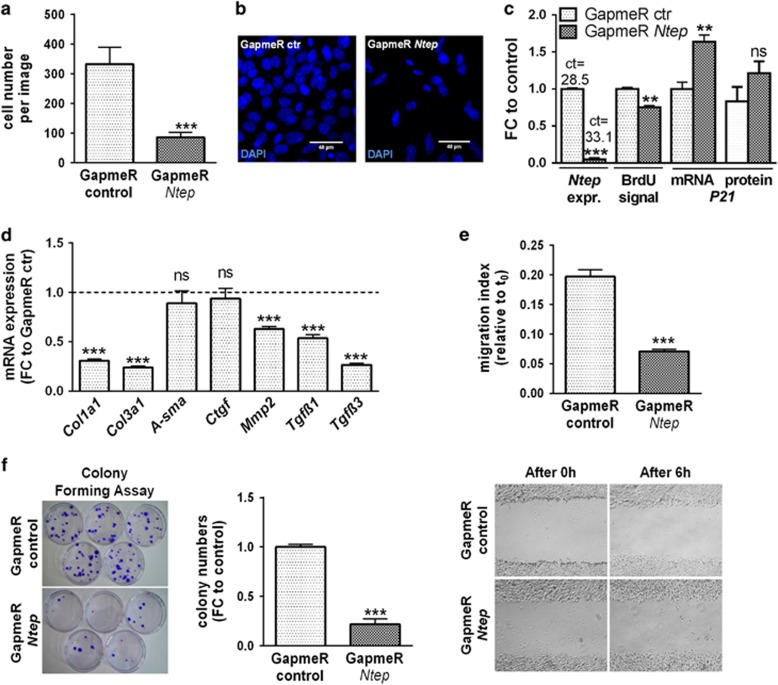
*Ntep* inhibition impairs fibroblast functions. (**a**) Cell number counted after treatment of 3T3 cells with GapmeR *Ntep* and GapmeR control. Six images per well taken, three wells per condition. (**b**) Example pictures of 4′,6-diamidino-2-phenylindole (DAPI)-stained 3T3 cells 48h after treatment with GapmeR *Ntep* and GapmeR control. (**c**) Expression level of *P21* mRNA, protein and *Ntep*, and level of proliferation of 3T3 cells after 48h of GapmeR *Ntep* treatment. Expression level was measured by qPCR. Ct values are given for *Ntep* expression above the respective bars. Protein level was assessed using western blotting. Proliferation rate was measured in BrdU enzyme-linked immunosorbent assays. (**d**) Expression level of *Col1a1, Col3a1, A-sma, Ctgf, Mmp2, Tgfβ1* and *Tgfβ3* mRNA in 3T3 cells treated with GapmeR *Ntep* and GapmeR control for 48 h. Expression level was measured with qPCR. (**e**) Example pictures of 3T3 cells treated with GapmeR *Ntep* or GapmeR control for 48 h and scratched to assay migration capacity. Pictures show time point 0 and 6 h after scratch wound was carried out. Migration index of 3T3 cells from three independent experiments. (**f**) Example picture of petri dishes seeded with single cells for colony-forming assay and stained with crystal violet. Cells were treated with GapmeR *Ntep* or GapmeR control 11 days before staining and 200 cells were seeded per Petri dish. Colony numbers of 3T3 cells from three independent experiments. All data are mean fold-change (FC) relative to control±S.E.M. (*n*=3 independent experiments), except stated otherwise. *P<0.05; **P<0.01; ***P<0.001. Student’s *t*-test

Silencing of *Ntep* had an impact on various aspects of fibroblast biology. In line with reduced proliferation rates, we found significantly less cells when *Ntep* was silenced. The remaining cells lost their usual elongated shape that enables contact to cells in the vicinity. Many cells showed an arched shape and their nuclei were smaller compared with control cells (Figures 3a and b). We confirmed this observation in BrdU incorporation assays. In parallel to *Ntep* transcript suppression (GapmeR control ct=28.5 *versus* GapmeR *Ntep* ct=33.1) and indicative of decreased proliferation rates, the BrdU signal was significantly lower (Figure 3c and Supplementary Figure S9A). In contrast, expression of the cell cycle regulator *P21* mRNA (*Cdkn1a*) was strongly increased in 3T3 cells transfected with GapmeR *Ntep* (Figure 3c and Supplementary Figure S9A). A similar trend (*P*=0.16) was observed for p21 protein levels. These findings prompted us to test whether the link between P21 and *Ntep* is also present *in vivo*. We measured *Ntep* in late generation telomerase knockout mice (G4), which are known to have elevated levels of P21 owing to high levels of telomeric DNA damage.^23^ In line with the *in vitro* data, late generation telomerase knockout mice expressed significantly less *Ntep* compared with early generation (G1) knockout and wild-type mice (both have long and intact telomeric DNA) (Supplementary Figure S10).

Fibroblasts produce and secrete proteins of the extracellular matrix and have a central role as mediators of pathological fibrosis and hence their pharmacological inhibition is a therapeutic strategy.^24, 25^ To test whether pharmacological inhibition of *Ntep* impairs the production of extracellular matrix, we measured mRNA levels of several fibroblast markers. *Ntep* inhibition significantly reduced the expression of collagen 1a1 (*Col1a1*), collagen 3a1 (*Col3a1*), matrix metallopeptidase 2 (*Mmp2*), transforming growth factor *β*1 (*Tgfβ1*) and transforming growth factor *β*3 (*Tgfβ3*), but not *α*-smooth muscle actin (*A-sma*) and connective tissue growth factor (*Ctgf*) (Figure 3d and Supplementary Figure S9C). We also found that the migration capacity in GapmeR *Ntep*-treated cells was significantly reduced (Figure 3e and Supplementary Figure S9B). The ability of 3T3 cells with silenced *Ntep* to grow from single cells was severely impaired as indicated by colony-forming assays (Figure 3f). Our data suggest that *Ntep* is an essential regulator of a wide range of fibroblast characteristics.

**Figure 4:**
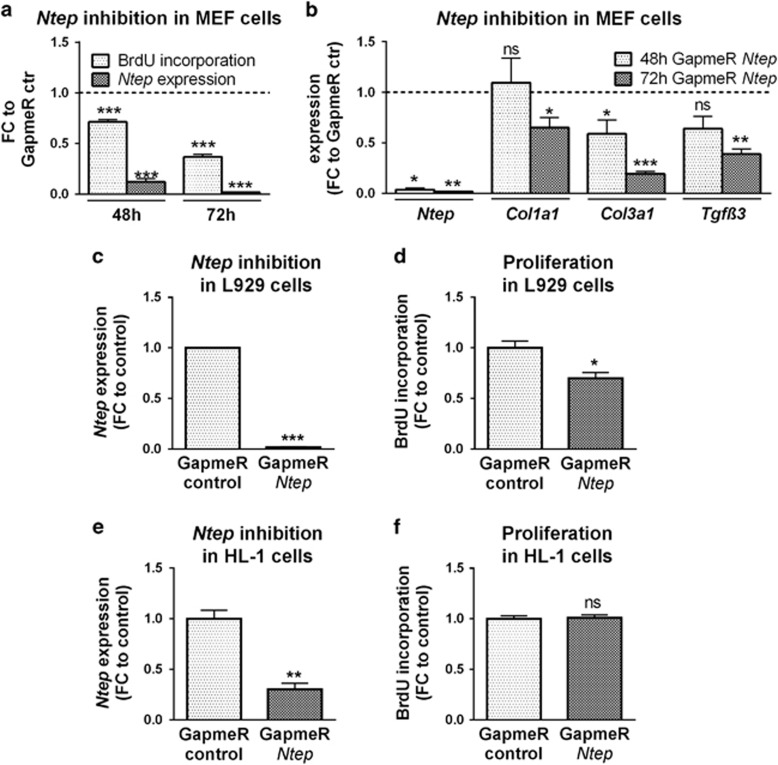
*Ntep* inhibition leads to decreased proliferation in murine fibroblasts, but not in cardiomyocytes. (**a**) Expression level of *Ntep* and proliferation rate of MEFs treated with GapmeR *Ntep* and GapmeR control for 48 and 72 h. Expression level was measured by qPCR. Proliferation rate was measured in BrdU enzyme-linked immunosorbent assays. (**b**) Expression level of *Ntep*, *Col1a1*, *Col3a1* and *Tgfβ3* mRNA after 48 h transfection of MEF cells with GapmeR *Ntep* and GapmeR control. Expression level was measured by qPCR. (**c**) Expression level of *Ntep* and (**d**) proliferation rate in L929 fibroblasts treated with GapmeR *Ntep* and GapmeR control for 48 h. Expression level was measured by qPCR. Proliferation rate was measured in BrdU enzyme-linked immunosorbent assays. (**e**) Expression level of *Ntep* and (**f**) proliferation rate in HL-1 cardiomyocytes treated with GapmeR *Ntep* and GapmeR control for 48 h. Expression level was measured by qPCR. Proliferation rate was measured in BrdU enzyme-linked immunosorbent assays. All data are mean fold-change (FC) relative to control±S.E.M. (*n*=3 independent experiments). *P<0.05; **P<0.01; ***P<0.001. Student’s *t*-test

To corroborate our findings in primary cells, we isolated mouse embryonic fibroblasts (MEFs) at E13.5 and tested the effects of *Ntep* knockdown. *Ntep* was efficiently silenced by GapmeR treatment. Importantly, *Ntep* inhibition also markedly decreased proliferation (Figure 4a) and the expression of collagens (*Col1a1*, *Col3a1*) and *Tgfβ3* (Figure 4b).

To test whether the effect of *Ntep* inhibition is limited to fibroblasts, we investigated *Ntep* inhibition in L929 cells and observed a similarly decreased proliferation (Figures 4c and d). In contrast, silencing *Ntep* in HL-1 cardiomyocytes (Figure 4e) did not affect proliferation (Figure 4f), suggesting that effects of *Ntep* inhibition may be fibroblast-specific. Finally, we asked whether increased expression of *Ntep* exerts opposite effects on fibroblasts. To this end, we generated stable lentiviral overexpression cell lines. Despite ~65-fold higher levels of *Ntep*, we did not observe significant effects when testing those cells in the assays outlined above (Supplementary Figure S11 and data not shown).

### Inhibition of *Ntep* stalls the cell cycle in G2/M and induces apoptosis

**Figure 5:**
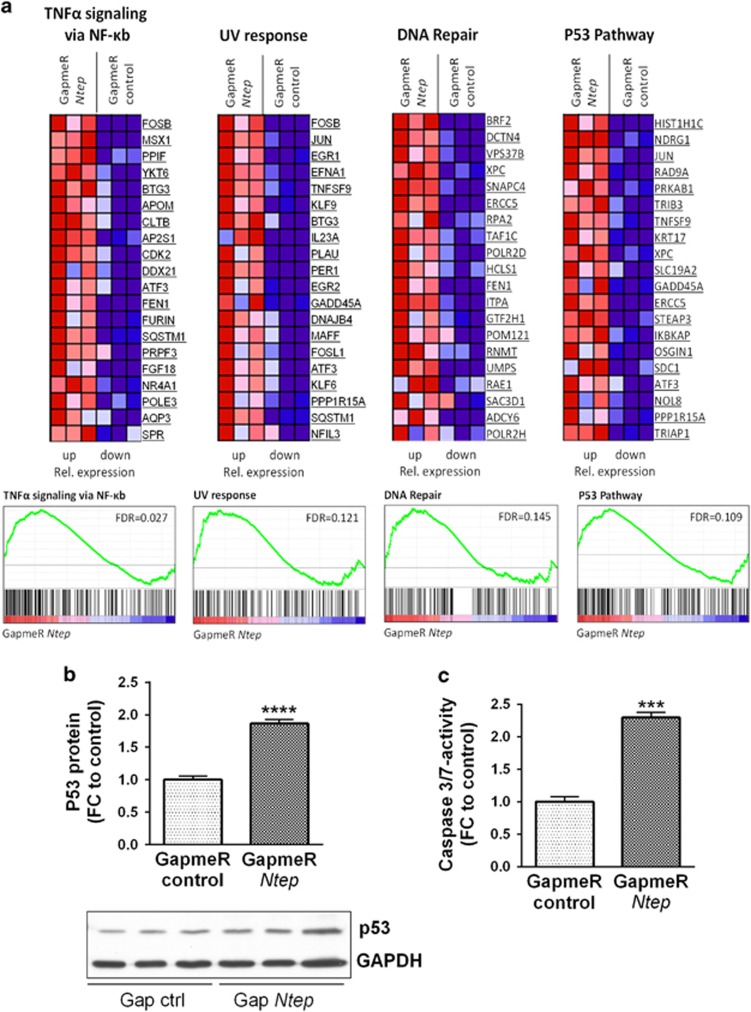
Microarray analysis reveals GapmeR-mediated P53 pathway activation. (**a**) Heatmaps of indicated gene sets with false discover rate (FDR) of <25% after GSEA showing the 20 most differentially expressed, core-enriched genes of 3T3 cells treated with GapmeR *Ntep* or GapmeR control. Matching GSEA plots for each pathway are shown beneath. Genes are ranked in GSEA plot according to expression level in the indicated sample. Genes on the left side are relatively highly expressed in GapmeR *Ntep* cells compared with controls, whereas genes on the opposite site are underrepresented. The red to blue horizontal bar represents the ranked list. According to the amount of genes enriched for each gene set, an enrichment score is calculated, which is shown by the green line in the GSEA plots. (**b**) Validation of P53 protein level in 3T3 cells treated with GapmeR *Ntep* and GapmeR control for 48 h. Expression level was measured using western blotting. Representative picture of a western blot showing the band of P21 and the housekeepeing gene GAPDH. (**c**) Caspase-3/7 activity after treatment of 3T3 cells with GapmeR *Ntep* and GapmeR control. Activity was measured using luminescent assays. All data are mean fold-change (FC) relative to control±S.E.M. (*n*=3 independent experiments). *P<0.05; **P<0.01; ***P<0.001. Student’s *t*-test

**Figure 6:**
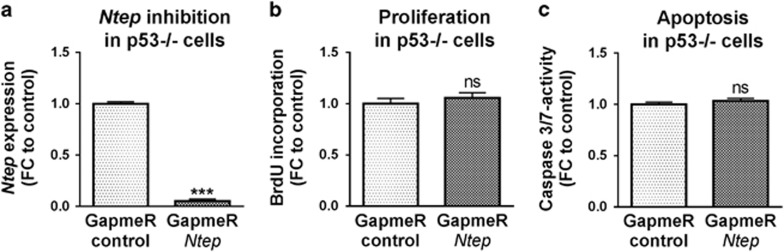
Effect of *Ntep* inhibition is not detectable in p53-deficient fibroblasts. (**a**) Expression level of *Ntep*, (**b**) proliferation rate of and (**c**) caspase-3/7 activity of p53-deficient primary MEF treated with GapmeR *Ntep* and GapmeR control for 48 h. Expression level was measured by qPCR. Proliferation rate was measured in BrdU enzyme-linked immunosorbent assays. Caspase activity was measured using luminescent assays. All data are mean fold-change (FC) relative to control±S.E.M. (*n*=3 independent experiments). *P<0.05; **P<0.01; ***P<0.001. Student’s *t*-test

To gain insight into the molecular mechanism of *Ntep* in fibroblast biology, we performed transcriptome profiling of 3T3 cells 48 h after *Ntep* silencing. Five hundred and nineteen genes were downregulated and 354 genes were upregulated in three independent experiments (fold-change cutoff: 2; *P*-value: <0.01) (Supplementary File 2). We performed gene set enrichment analysis (GSEA) as described^26, 27^ using hallmark gene sets to focus on specific, well-defined biological processes to reduce noise and redundancy (Supplementary Table S2). Gene sets associated with proliferation (e.g. mTORC1 signalling and c-myc targets) and DNA-damage pathways (UV response pathway, unfolded protein response pathway, DNA repair and TNF*α* signalling pathway) were significantly enriched (Figure 5a). In addition, the P53 pathway was significantly upregulated in response to *Ntep* silencing (Figure 5a). Higher levels of P53 in GapmeR *Ntep*-treated cells confirmed the microarray data (Figure 5b). Since apoptosis-related pathways were upregulated, we next measured caspase-3/7 activity. We found a marked increase in cells treated with GapmeR *Ntep* compared with GapmeR control (Figure 5c). To test whether the observed effects are directly mediated through P53, we repeated the experiments in *p53*-deficient fibroblasts. Strikingly, the effects on proliferation and apoptosis were completely rescued (Figures 6a–c), indicative of a direct *Ntep*–P53 axis.

**Figure 7:**
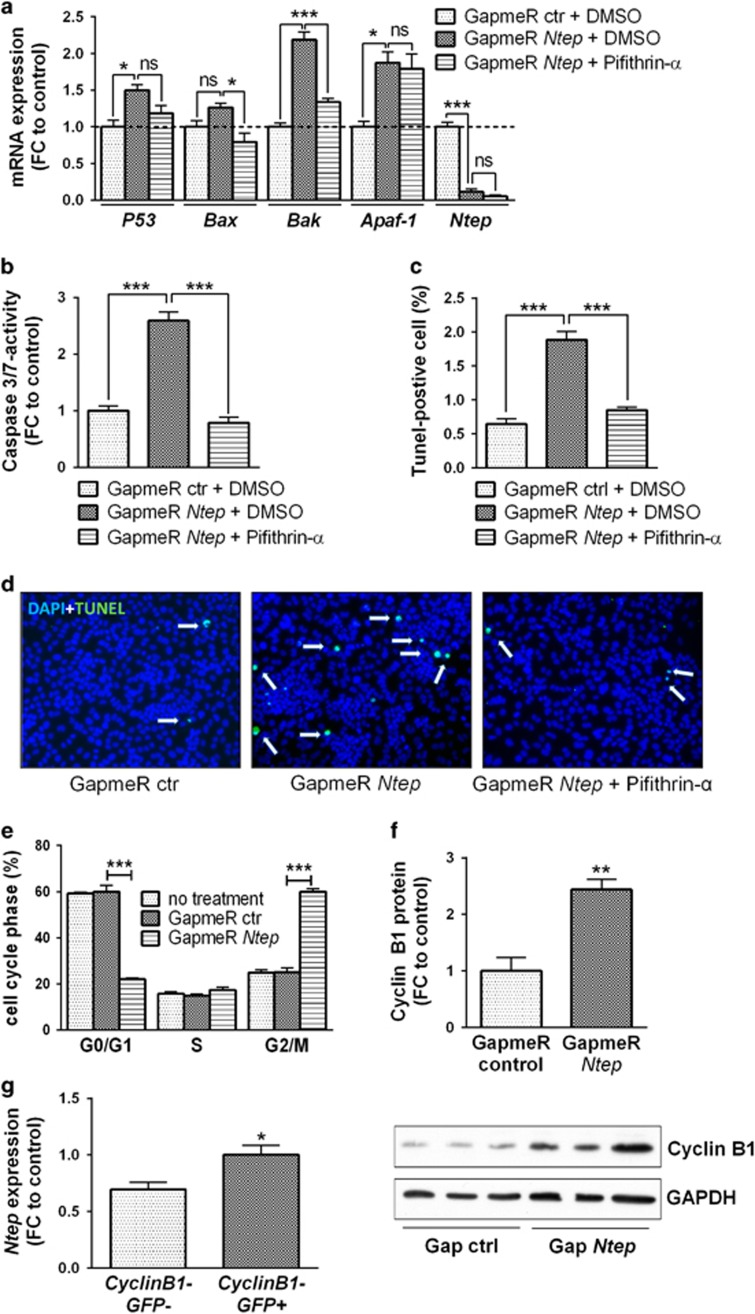
*Ntep* is essential for successful progression through the cell cycle. (**a**) Expression level of *P53*, *Apaf-1, Bax, Bak* mRNA and *Ntep* in 3T3 cells treated with GapmeR control and dimethyl sulphoxide (DMSO) and GapmeR *Ntep* and DMSO/pifithrin-*α*. Expression level was measured with qPCR. (**b**) Caspase-3/7 activity after treatment of 3T3 cells with GapmeR control and DMSO and GapmeR *Ntep* and DMSO/pifithrin-*α*. Activity was measured using luminescent assays. (**c**) TUNEL-positive cells after treatment of 3T3 cells with GapmeR control and DMSO and GapmeR *Ntep* and DMSO/pifithrin-*α*. TUNEL-positive cells were counted and normalized to 4′,6-diamidino-2-phenylindole (DAPI) signal. (**d**) Representative pictures from DAPI/TUNEL staining of 3T3 cells treated with GapmeR control and DMSO and GapmeR *Ntep* and DMSO/pifithrin-*α*. (**e**) Propidium iodide staining of 3T3 cells treated for 48 h with GapmeR *Ntep* and GapmeR control to analyse the cell cycle using fluorescence-activated cell sorter (FACS). Plots showing % of cells in the G0/G1 phase, cells in the S phase and cells in the G2/M phase. (**f**) Cyclin B1 protein level in 3T3 cells treated with GapmeR *Ntep* and GapmeR control for 48 h. Expression level was measured using western blotting. Representative picture of a western blot showing the band of cyclin B1 and the housekeeping gene GAPDH. (**g**) Expression level of *Ntep* in 3T3 cells stably expressing cyclin B1 coupled with GFP. Cells were sorted according to GFP signal in GFP-positive and GFP-negative cells. Expression level was measured with qPCR. *P<0.05; **P<0.01; ***P<0.001. Student’s *t*-test or one-way anaylsis of variance (ANOVA) for three groups. All data are mean fold-change (FC) relative to control±S.E.M. (*n*=3 independent experiments)

We then tested whether upregulation of P53 and caspase-3/7 activity can be blocked by pifithrin-*α*, an agent that suppresses DNA-damage-induced apoptosis by targeting targets of P53 but not P53 itself.^28, 29^ As expected, the mRNA level of *P53* was not significantly changed, but the level of its proapoptotic targets *Bak and Bax* dropped significantly This was not observed for the apotosome gene *Apaf-1*. PFT-*α* had no impact on the expression level of *Ntep* itself (Figure 7a). The activity of caspase-3/7 was fully rescued after PFT-*α* treatment (Figure 7b). These findings were confirmed by terminal deoxynucleotidyl transferase dUTP nick-end labelling (TUNEL) staining, inhibition of *Ntep* significantly increased the number of TUNEL-positive cells, which was rescued by PFT-*α* treatment (Figures 7c and d).

We next investigated which cell cycle check point is affected by *Ntep* inhibition. At 48h or 72 h after transfection with GapmeR *Ntep* or controls, 3T3 cells were stained with propidium iodide followed by cell cycle analysis. No differences were observed during the S phase. However, *Ntep* knockdown resulted in a marked drop of cells in G0/G1, whereas the number of cells residing in the G2/M phase was doubled (Figure 7e and Supplementary Figure S12). The level of G2/M-specific cyclin B1 was strongly increased after inhibition of *Ntep* (Figure 7f), suggesting that the cells are stalled in G2/M. In addition, we used a cyclin B1-GFP reporter^30^ to FACS-sort 3T3 cells into GFP-positive and GFP-negative cells. In line with the former results, the expression of *Ntep* was significantly higher in the S/G2/M phase (GFP-positive fraction) compared with cells in the G1 phase (GFP-negative fraction) (Figure 7g). To test whether the transition from M to G1 is impaired, we arrested cells with nocodazol in the M phase. However, after releasing this cell cycle blockade, we did not observe differences between GapmeR *Ntep-*treated and control cells (data not shown). Collectively, these data suggest that *Ntep* is regulated in a cell cycle-dependent manner and that it is required for the progression through the G2/M phase of the cell cycle.

### *Ntep* is a conserved lncRNA

Finally, as antifibrotic targets for treatment of human disease (cardiac-, lung-, kidney fibrosis, etc.) are scarce and non-coding RNAs are emerging as novel, druggable disease targets,^31^ we investigated if homologues of *Ntep* can be identified in other species including human. To this end, we searched the human genome assembly (GRCh38.p10) and rat genome assembly (Rnor_6.0) from Ensembl for putative homologues in human and rat. The sequence of *Ntep* was blasted against the sub-database 'Ensemble Non-coding RNA genes'. Two recently annotated non-coding transcripts, ENST00000344893 and ENSRNOT00000079320 were identified as the best homologues of *Ntep* in the human and rat genome, which represent a remarkable degree of sequence conservation of 42.9% and 37.7%, respectively (Supplementary Figure S13).

## Discussion

LncRNAs are emerging novel regulators of various cellular mechanisms.^32^ In fact, owing to the large number of lncRNAs and their diverse modes of action, it is likely that they have crucial roles in virtually any cellular pathway.^4^ To discover pathway-specific lncRNAs several methods such as qPCR-profiling platforms, microarray or next-generation sequencing (NGS)-based methods are available. Nevertheless, owing to the relatively low expression of many lncRNAs, NGS requires a high sequencing depth (70–300 million reads), which comes at high costs and only gives information about lncRNA abundance but not function.

Here, we describe the development and application of a large 26 k shRNA library approach for the unbiased discovery of lncRNA functions, which is customizable and suitable for other screening applications. In this study, the inhibition of lncRNAs with shRNAs was combined with the readout of the shRNA barcode via NGS. This provides a low level of background noise, because virtually every shRNA is detected. Importantly, our library contains six to seven different shRNAs for each lncRNA target (in total 3842 non-coding targets, incl. lncRNAs and UCE), which largely enhances lncRNA knockdown efficiency and simultaneously limits off-target effects. In particular, we considered lncRNAs only a 'hit' if in the screening approach at least four out of the six to seven shRNAs for a given lncRNA were identified.

Thus, we report a method that combines intelligent loss-of-function screening by RNA interference (RNAi) with the advantages of NGS to screen for immediate identification of specific lncRNA functions.

To provide proof of concept, we applied our library to 3T3 fibroblasts and aimed to identify lncRNAs, which have crucial roles in fibroblast proliferation. We identified and further validated one particular lncRNA, which we named *Ntep*.

Functionally, we found that *Ntep* expression is decreased in antiproliferative environments such as hypoxia and starvation. With cycle threshold (ct) values of ~28.5 in 3T3 cells, *Ntep* is a relatively abundant lncRNA and its silencing by GapmeRs inhibits proliferation and strongly impairs common fibroblast features. This was also true for other fibroblasts such as MEF cells and L929 fibroblasts, but not for HL-1 cardiomyocytes, suggesting a rather fibroblast-specific function. Fibroblasts lacking *Ntep* lose their ability to migrate and to express major markers of fibroblasts. Our initial screening identified *Ntep* as a regulator of proliferation, after which we named this uncharacterized lncRNA. However, our in-depth analyses suggests that loss of *Ntep* may have more global effects and affects cell viability of fibroblasts, whereas *Ntep* seem dispensable in other cell types (HL-1). In contrast to the antiproliferative and antifibrotic consequences of *Ntep* inhibition, we do not find evidence for opposite effects when *Ntep* was overexpressed. LncRNA overexpression using lentiviral constructs does not necessarily increase the endogenous function of the target, as it is critical where the overexpressed transcript locates to and whether the native three-dimensional structure can be formed. An attempt to mimic the natural function harbours challenges, which has already been reported for several studies when overexpressing lncRNAs.^33^

Mechanistically, we demonstrated that *Ntep* is cell cycle regulated. *Ntep* levels in the G1 phase are significantly lower than in subsequent phases, which is in line with the finding that cells depleted for *Ntep* arrest in the G2/M phase of the cell cycle and express higher levels of cyclin B1. Cells lacking *Ntep* seem to pass the first cell cycle checkpoints but do not enter into or do not complete mitosis. This may explain the abnormal morphology of nuclei from GapmeR *Ntep*-treated cells, which appear shrunken and have a rather irregular shape. The GSEA data revealed that several pathways associated with DNA-damage responses are upregulated after *Ntep* inhibition including 'the P53 pathway', 'the upregulation of UV response' and 'increased DNA repair'. This suggests that lack of *Ntep* results in a higher load of DNA damage. Accordingly, we identified increased levels of apoptosis in those cells concomitant with a higher expression of the proapoptotic factors *Bax* and *Bak*, as well as *Apaf-1* as a component of the apoptosome.^34^

We also found significantly higher expression of P53 upon inhibition of *Ntep*. Interestingly, increased level of apoptosis can be rescued by treatment with PFT-*α*, which inhibits P53 targets indicating that *Ntep* does not directly repress proapoptotic genes, but is rather associated with the master regulator P53. This was further supported by a rescue of the proliferation and apoptosis phenotypes when *Ntep* was inhibited in p53-deficient fibroblasts. Our data further suggest that also P53 activation is an effect secondary to a stalled cell cycle and higher level of DNA damage (as shown by upregulated DNA-damage response pathways).

In summary, this study provides proof of principle for a comprehensive large shRNA library approach for functional identification of lncRNAs important for proliferation. This approach is customizable to other screening settings, like pathway analysis through combination with biosensors (i.e. fluorophore-labelled 'protein X'). Subsequent molecular analysis revealed that *Ntep* is cell cycle regulated and indispensable for completing the cell cycle and maintaining the fibroblast phenotype of 3T3 cells, and also primary fibroblasts. Finally, the identification of a human homologue transcript warrants further investigation of the role of *Ntep* in fibrosis biogenesis and potential therapeutic target.

## Materials and methods

### Animal study

All animal studies were performed in accordance with the relevant guidelines and regulations and with the approval of the Niedersächsisches Landesamt für Verbraucherschutz und Lebensmittelsicherheit.

The tissue for measuring the expression of *Ntep* in different organs was harvested from 12-week-old C57BL6J male mice.

### Statistics

All *in vitro* experiments were performed as indicated in the corresponding figure legends; in general, three independent experiments were performed (*n*=3). For each independent experiment, three biological replicates were generated, unless stated otherwise. Thus, the statistics were calculated using the mean value of each experiment. Data are presented as mean of independent experiments/independent samples±S.E.M. The variances were similar between groups that are being compared. Statistical analysis was carried out using GraphPad Prism 6 (GraphPad Software, La Jolla, CA, USA). For analysis of two groups, an unpaired two-tailed Student’s *t*-test was used. For comparison of three or more groups, one-way ANOVA followed by Tukey’s post-test was applied.

### Cell culture experiments

Mouse fibroblast 3T3 cells, MEFs, p53-deficient primary MEFs (a kind gift from Dr Natalia Ronkina, Institute of Cell Biochemistry, Hannover Medical School, Hannover, Germany) and HEK293T cells were cultured in DMEM (Thermo Fisher Scientific, Waltham, MA, USA) supplemented with 10% FBS (Sigma-Aldrich, St. Louis, MO, USA) and 1% penicillin/streptomycin (100 U/ml : 100 *μ*g/ml; Sigma-Aldrich) at 37 °C in 5% CO_2_. L929 fibroblasts were cultured in DMEM supplemented with 10% FBS, 1% penicillin–streptomycin and 2mM l-glutamine. HL-1 murine cardiomyocytes were cultured in claycomb medium (Sigma-Aldrich) supplemented with 10% FBS, 1% penicillin–streptomycin, 0.1 mM norepinephrine and 2mM K-glutamine. Primary MEFs were isolated from E13.5 embryos using standard methods from C57BL/6N mice. MEF cells were used for experiments until the fifth passage. For post-transcriptional lncRNA silencing, GapmeRs (Exiqon part of Qiagen, Venlo, The Netherlands) against *Ntep* were used. Cells were transiently transfected using 50 nM GapmeRs and X-tremeGENE HP Transfection Reagent (Sigma-Aldrich). GapmeR-negative control a (=GapmeR control) was used for control treatment (5′-AACACGTCTATACGC-3′). Three different *Ntep* sequences were tested initially (GapmeR *Ntep* no. 1: 5′-TTACGGCTGTCTTCTT-3′ GapmeR *Ntep* no. 2: 5′-TACATCACTTCATAGG-3′ GapmeR *Ntep* no. 3: 5′-TCTGGAGTTAGTCGTT-3′). The inhibition was strongest using GapmeR *Ntep* no. 3, which we call GapmeR *Ntep* in the manuscript. Inhibition of P53 targets was achieved by treating cells with 10 *μ*M pifithrin-*α* (Enzo Life Sciences, Farmingdale, NY, USA) solved in DMSO. For hypoxia studies, 3T3 cells were exposed to low oxygen (0.2% O_2_) for 24, 48 and 72 h. For induction of starvation, 3T3 cells were grown for 24 h in DMEM supplemented with 1% penicillin–streptomycin and 1% FBS or 0.1% FBS. The cells lines used in this study are tested for mycoplasma contamination on a regular basis (every 1–2 months). 3T3 cells are from the lab of Stefan Engelhardt (TU München, Germany), and HEK293T cells are from the lab of Nico Lachmann (MH Hannover, Germany).

### RNA isolation

Total RNA of tissues and cultured cells was isolated using RNeasy Mini Kit (Qiagen) or TriFast method (VWR Life Science, Radnor, PA, USA) according to the manufacturer’s instructions. Quantification and quality control were performed with Synergy HT Reader (BioTek Instruments, Winooski, VT, USA).

### Reverse transcription

For gene expression analysis, 100–1000 ng RNA was reverse transcribed with iScript Select cDNA Synthesis Kit (Bio-Rad Laboratories, Hercules, CA, USA). For gene-specific reverse transcription, Superscript III Reverse Transcriptase (Thermo Fisher Scientific) was used.

### DNase digest/*Ntep* amplicon validation

For detection in subcellular fractions and for validation of the *Ntep* primers, total RNA was DNase-treated with the RNase-free DNase set (Qiagen) before reverse transcription. Five hundred nanogram of RNA was incubated with 0.3 U of DNase I in 20 *μ*l total volume, containing 1x RDD buffer (Qiagen) and 10 U RNAseOUT (Thermo Fisher Scientific), at 37 °C for 30 min. The digestion was stopped by adding 1.25 mM EDTA and incubated at 65 °C for 5 min. For reverse transcription up to 10 *μ*l DNase-digested RNA were used. The removal of the DNA was confirmed by performing reverse transcription in the absence and presence of reverse transcriptase (±RT) before GS-PCR or qPCR. The amplicon from qPCR and GS-PCR was separated on a 2% agarose gel, excised and the QIAquick Gel Extraction Kit (Qiagen) was used to extract and to clean the DNA fragment. The sequence was validated using by DNA sequencing (Eurofins Scientific, Luxembourg).

### qPCR analysis

For quantitative detection of lncRNAs and mRNAs, the cDNA was used for qRT-PCR in a CFX96 Touch Real-Time PCR Detection System (Bio-Rad) with specific primers (Supplementary Table S1) and the iQ SYBR Green Mix (Bio-Rad) according to the manufacturer’s protocol.

Gene expression levels were normalized to levels of hypoxanthine-guanine phosphoribosyltransferase, 18S ribosomal RNA or *β*-actin. In addition to relative expression, in some figures, the expression of *Ntep* is given as 'ct value'.

### Polymerase chain reaction

For amplification of *Ntep* the HotStarTaq Master Mix Kit (Qiagen) was used according to the manufacturer’s protocol.

### Genome-wide pooled shRNA screen

A 26 391 element shRNA library (26 k) was created to target 3842 murine lncRNAs and ultraconserved elements. The targets were chosen from lncRNAs listed in RefSeq (https://www.ncbi.nlm.nih.gov/refseq/) in 2013 (Cellecta, Mountain View, CA, USA), from the top 50 hits from microarray lncRNA analysis of Viereck *et al.*^35^ and unpublished data and from lists of ultraconserved elements.^36^ The barcoded shRNA sequences were assembled into a pRSI16 vector backbone, containing a puromycin resistance marker and an RFP reporter (Supplementary Figure S1). The transduction efficiency of 3T3s cells was determined measuring the percentage of RFP-positive cells at the core facility cell sorting (Hannover Medical School). The shRNA expression was under the control of a constitutive U6 promotor and each shRNA was linked to a unique barcode, which could be identified by HT sequencing. The library contained 6–7 shRNAs per lncRNA and additionally 38 shRNAs to target luciferase as an internal control. The threshold for analysing the results was set according to the luciferase shRNA distribution. Twenty million 3T3 cells were infected with an MOI of 0.5, which allows an infection rate of at least 100 cells per shRNA. The infection was carried out in suspension using polybrene (1 *μ*g/ml; Santa Cruz, Dallas, TX, USA). After 3 days, the cells were selected using puromycin (5 *μ*g/ml; Santa Cruz) for 2 days. In advance, the concentration of puromycin was tested in a dose–response range from 0.5 to 10 *μ*g/ml, to identify the concentration that would destroy >90% of cells in 72 h. After puromycin selection, an aliquot of the cells was taken for baseline sampling for sequencing. Five million infected cells were stained with CFSE according to the manufacturer’s description (CellTrace CFSE Cell Proliferation Kit Protocol; Thermo Fisher Scientific). Additionally, an aliquot of non-infected 3T3 cells was stained. The cells were allowed to proliferate for 5 days. Afterwards, the proliferative status of the 3T3 cells was measured using the FACS Aria Fusion System (BD Bioscience, Franklin Lakes, NJ, USA) at the core facility cell sorting (Hannover Medical School). The sorting gates were set according to the signal of the non-infected, but CFSE-stained 3T3 cells, assuming that the library-infected, CFSE-stained 3T3 cells would have a different proliferative status. Infected cells with a high and low CFSE signal were sorted (two populations) (Supplementary Figure S2). Together with the baseline sampling aliquot, the samples were sent to Cellecta for further processing. DNA was extracted and the harbouring barcodes amplified. The representation of the barcodes was measured by HT sequencing on an Illumina GA2X System (Illumina, San Diego, CA, USA). The complete raw data can be found in Supplementary File 1. The labels in brackets refer to the labels of Supplementary File 1. The plasmid collection used for shRNA library generation was sequenced ('plasmid') and normalized to 20 million reads ('plasmid_20M'). Further, the baseline sample, which was taken from the 3T3 cells infected with the shRNA library after puromycin selection before CFSE staining, was sequenced ('baseline') and normalized to 20 million reads ('baseline_20M'). These two normalized values were compared to secure an even shRNA distribution before and after transduction of the initial library (Figure 1b). The sequencing results derived from cells with a weak ('CFSE-low') or strong CFSE ('CFSE-high') signal were normalized to 20 million reads as well ('_20M'). The shRNA distribution was calculated as the ration of 'CFSE-low_20M' or 'CFSE-high_20M' to 'baseline_20M'. To buffer random count variations in low-abundance shRNAs, a small fixed amount of reads was added to each value before calculating the ratio ('+20'). Further, the distribution of shRNAs against a luciferase control was measured in CFSE-low/high cells. Since this distribution is random, it was used as a cutoff for random shRNA distribution. The final shRNA distribution in cells with weak ('CFSE-low') or strong CFSE ('CFSE-high') signal is given as 'Norm (Ratio CFSE-low/baseline)' and 'Norm (Ratio CFSE-high/baseline)'. We wanted to focus on lncRNAs whose inhibition was leading to a decrease in cell proliferation. Thus, we searched for shRNAs that were overrepresented in the population with a high CFSE signal (low proliferating cell pool) and underrepresented in the population with a low CFSE signal (high proliferating cell pool). Normalized barcode reads in the sub-population with a CFSE signal higher than fold-change=1.3 were considered, in the sub-population of a low CFSE signal the read was considered when it was lower than 0.7.

### Generation of transgenic 3T3 cell lines

#### 3T3 cells stably expressing cyclin B1-GFP

The plasmid used for generation of 3T3 cells stably overexpressing cyclin B1 fused to GFP was a kind gift from Amir Eden's lab.^30^ Lentiviral particles were produced in HEK293T cells with the help of additional plasmids pMDL-g/pIIE, pRSV-rev and pCMV-VSVG using liposomal transfection. The supernatant was harvested 48 and 72 h post-transfection and concentrated applying Amicon Ultra Centrifugal Filter Units (Merck Millipore, Billerica, MA, USA) with a molecular weight cutoff of 100 000. Concentrated lentivirus was transduced to 3T3 cells at ratios from 1 : 100 up to 1 : 20 000 to calculate the viral titre. 3T3 cells were infected with the viral particles using protamine sulphate. 3T3 cells+pLV− cyclin B1-GFP were sorted according to the GFP signal using the FACS Aria Fusion System (BD Bioscience) at the core facility cell sorting (Hannover Medical School).

#### 3T3 cells stably expressing *Ntep*

For overexpression of *Ntep* in combination with a selectable marker, bidirectional lentiviral vectors (pLV+) containing an antisense-oriented expression unit for *Ntep* (NR_015491.1.1) and a sense-oriented expression unit for eGFP-2A-Puro were cloned. Vectors lacking the *Ntep* sequence were used as controls (pLV+empty). Lentiviral particles were produced in HEK293T cells with the help of additional plasmids pMDL-g/pIIE, pRSV-rev and pCMV-VSVG by transient transfection according to standard procedures.^37^ Viral supernatants were harvested 48 h post-transfection and transduced into 3T3 cells. After the initial puromycin selection, transgenic cells were cultured as described above.

### Transcriptome profiling after *Ntep* suppression

Total RNA was isolated from 3T3 cells treated with GapmeR *Ntep* and GapmeR control for 48 h using RNeasy Mini Kit (Qiagen) from three independent experiments. Agilent 2100 Bioanalyzer (Agilent Technologies, Santa Clara, CA, USA) was used for RNA quantification and quality control before microarray-based mRNA. Microarray raw data (Mouse GE 4x180K; Agilent Technologies) used or referred to in this publication were generated by the Research Core Unit Transcriptomics of Hannover Medical School.

To identify genes whose expression is influenced by *Ntep* levels in fibroblasts, a GSEA was performed.^26, 27^ GSEA was applied using annotations from hallmark gene set to gain reduced noise and redundancy.^38^ Thirteen gene sets were reported to be upregulated in *Ntep*-suppressed samples, of which eight gene sets were selected with an FDR threshold of below 25% as suggested by the GSEA User Guide from Broad Institute. The complete list is shown in Supplementary Table S2.

### Subcellular fractionation

Fragmentation of 3T3 cells into cytoplasmic and nucleic fractions was performed as described previously.^39^

### Proliferation assay

For cell proliferation assays, 3T3, MEF, HL-1 and L929 cells were seeded into 96-well plates. BrdU Cell Proliferation ELISA Kit (Abcam, Cambridge, UK) was used to measure the proliferation rate of fibroblasts. The assay was performed according to the manufacturer’s instructions.

### Colony-forming assay

3T3 cells were treated with either GapmeR *Ntep* or GapmeR control as described above and harvested 48 h post-transfection. To measure the ability of single cells to grow in a colony, 100 mm Petri dishes were seeded with 200 cells each. Colonies were monitored over a period of 11 days, after that the colonies were fixed, stained with crystal violet (0.5% (w/v); Sigma-Aldrich) and counted.^40^ The assay was carried out in three independent experiments. For each experiment, three biological replicates were performed with five Petri dishes per replicate.

### Cell cycle analysis

After 48 and 72 h of transfection of 3T3 cells with either GapmeR *Ntep* or GapmeR control as described above, cells were fixed and stained for cell cycle analysis using Guava Cell Cycle Reagent for Flow Cytometry (Merck Millipore) according to the manufacturer’s instructions.

### Scratch assay

The migration capacity of 3T3 cells with a suppressed level of *Ntep* was monitored. Therefore, 3T3 cells were transfected with either GapmeR *Ntep* or GapmeR control. Three hours before scratching, the cells were treated with mitomycin C (10 *μ*g/ml; Sigma-Aldrich) to stop proliferation and a horizontal scratch through the well was made using a pipette tip. Microscopic images were captured 0, 4, 6 and 24 h after the scratch application. The wound area was calculated by the use of NIS-elements BR Software (Nikon, Tokio, Japan). Migration index was calculated using the formula: (area (0 h)−area (6 h))/area (0 h).

### Caspase-3/7 assay

The activation of caspase-3/7 was measured in GapmeR *Ntep* or GapmeR control-treated 3T3 cells using the Caspase-Glo 3/7 Assay Systems (Promega, Fitchburg, WI, USA) according to the manufacturer’s instructions.

### TUNEL staining

TUNEL was performed in GapmeR *Ntep*- or GapmeR control-treated 3T3 cells using the *In Situ* Cell Death Detection Kit Fluorescein (Sigma-Aldrich). Three repeats per group were performed with 15 images taken and analysed for each repeat. The TUNEL-positive cells and DAPI-positive cells were counted from each image for analysis.

### Protein coding potential

To assess the protein coding potential of *Ntep* potential, ORFs were calculated using the given sequence for *Ntep* from RefSeq (Supplementary Figure S6). We used two independent tools to enhance the quality of predictions; Sequence Manipulation Site: ORF Finder^21^ and Coding Potential Calculator.^22^ To check 3T3 cells for potential peptides arising from the ORF, 3T3 cells stably overexpressing *Ntep* (3T3 pLV+*Ntep*) or harbouring a control vector (3T3 pLV+empty) were collected and the protein content isolated according to standard conditions. The protein lysate were alkylated using acrylamide (40%, 4K solution; Applichem, Darmstadt, Germany) for the purpose of mass spectrometry analysis. Thirty micrograms of each lysate were loaded onto 4–15% Mini-PROTEAN TGX Precast Protein Gels (Bio-Rad) and separated using SDS-PAGE. Proteins were stained using Coomassie Brillant Blue G250 (Thermo Fisher Scientific) according to standard procedure. The stained SDS-gel was further processed for mass spectrometry analysis at the MS Core Facility Proteomics at the Hannover Medical School. Therefore, the proteins samples in the designated range (Supplementary Figure S6) were in-gel digested and the harbouring peptides were analysed using the LC-MS System (Thermo Fisher Scientific, Waltham, MA, USA). Resulting data were searched for the predicted peptides against an in-house database.

### Western blotting

A total of 10–30 *μ*g of total protein were separated by SDS-PAGE, transferred to PVDF membrane and analysed by western blotting using standard protocols. The following antibodies were used to detect antigens: cyclin B1 (Santa Cruz; sc-245), p53 (Cell Signaling; 2524s), p21 (Thermo Fisher Scientific; MA1-91045) and GAPDH (Abcam; ab8245). Luminol reagent was used to detect the signals on the membrane using X-ray films (Kodak, Nusew York, Vereinigte Staaten, USA). Band intensity was calculated using the ImageJ Software (NIH, Bethesda, MD, USA).

### Fluorescence confocal microscopy

For immunofluorescence confocal microscopy, cells treated with GapmeR *Ntep* and GapmeR control were fixed in 4% PFA and permeabilized with 0.1% Triton X-100, followed by washing with PBS. Cells were stained with DAPI (1:1000; Sigma-Aldrich) in 5% donkey serum in PBS for 60 min. After washing with PBS, slides were embedded with Prolong Antifade (Thermo Fisher Scientific). Confocal imaging was prepared with a Zeiss LSM 780 using a Plan-Apochromat x40 water objective (Zeiss, Oberkochen, Germany).

## Accession number

The microarray data set can be accessed under the GEO accession number GSE94049; https://www.ncbi.nlm.nih.gov/geo/query/acc.cgi?acc=GSE94049.
