# Supplementary material for: A large shRNA library approach identifies lncRNA Ntep as an essential regulator of cell proliferation
Source: Cell Death Differ. 2017 Nov 3;25(2):307–18. doi: 10.1038/cdd.2017.158 (PMC5762845; doi:10.1038/cdd.2017.158)
Supplement: Supplementary Figures and Tables [file cdd2017158x1.ppt]

## Slide 1
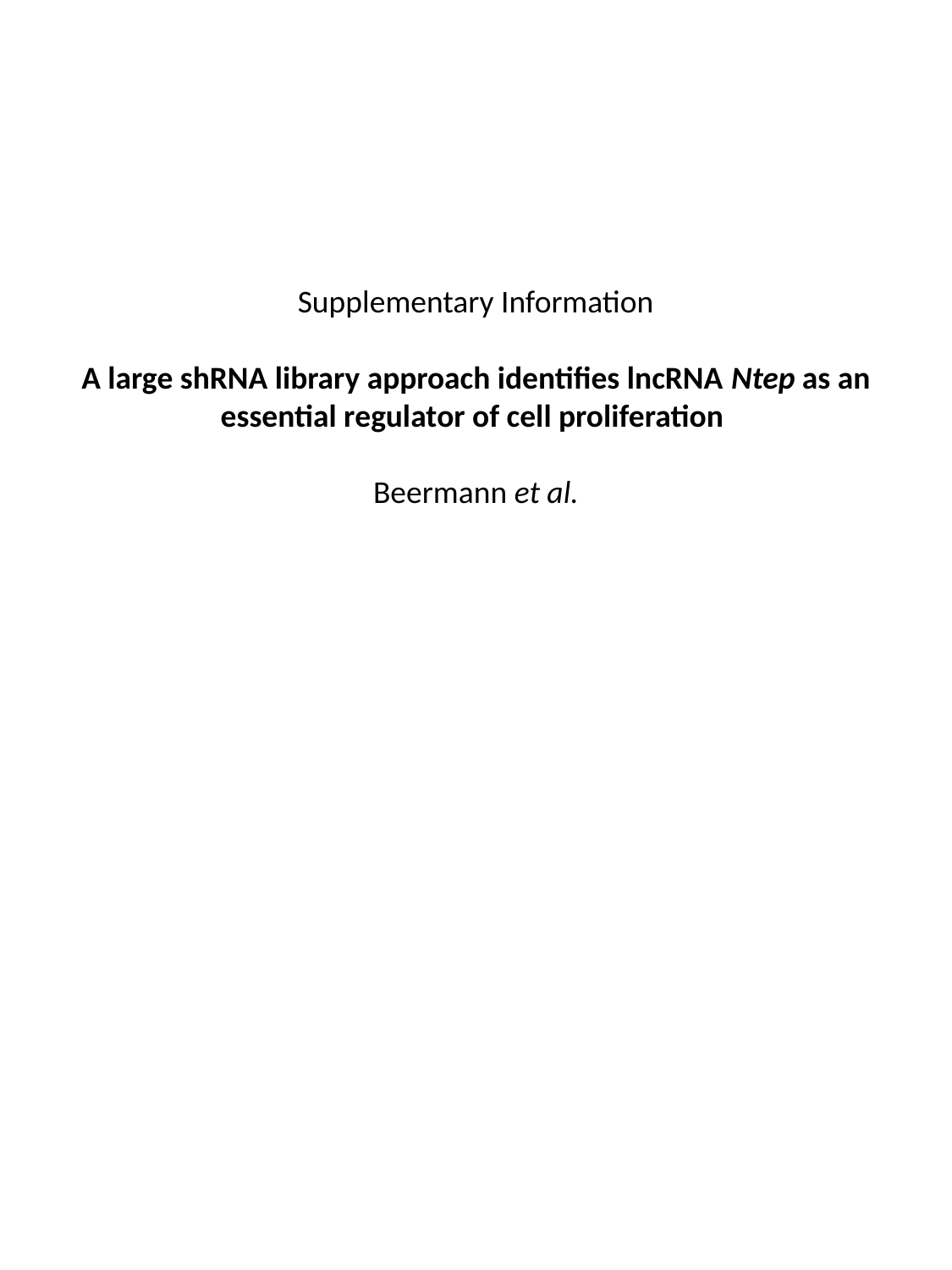

Supplementary Information
A large shRNA library approach identifies lncRNA Ntep as an essential regulator of cell proliferation
Beermann et al.

## Slide 2
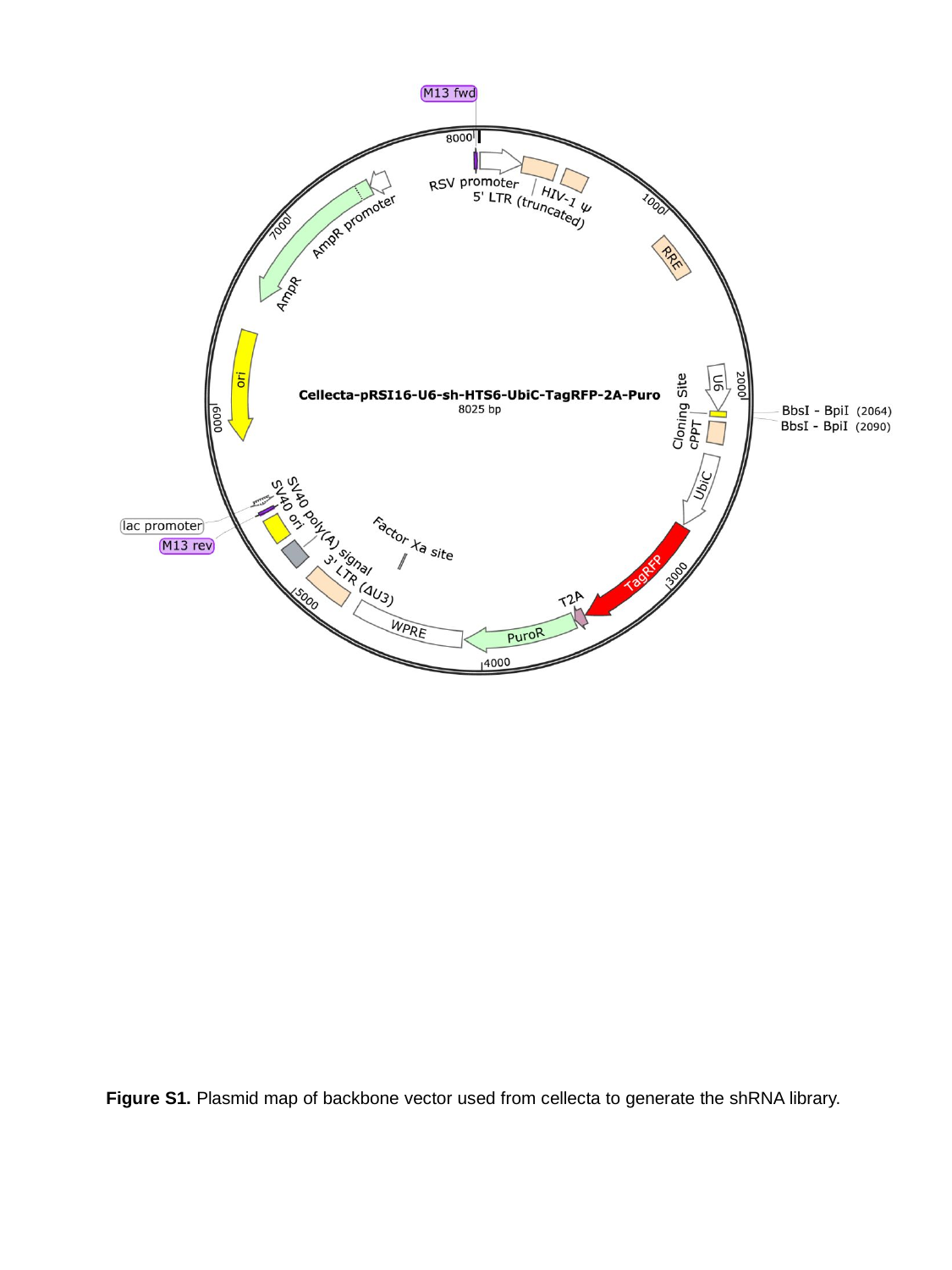

Figure S1. Plasmid map of backbone vector used from cellecta to generate the shRNA library.

## Slide 3
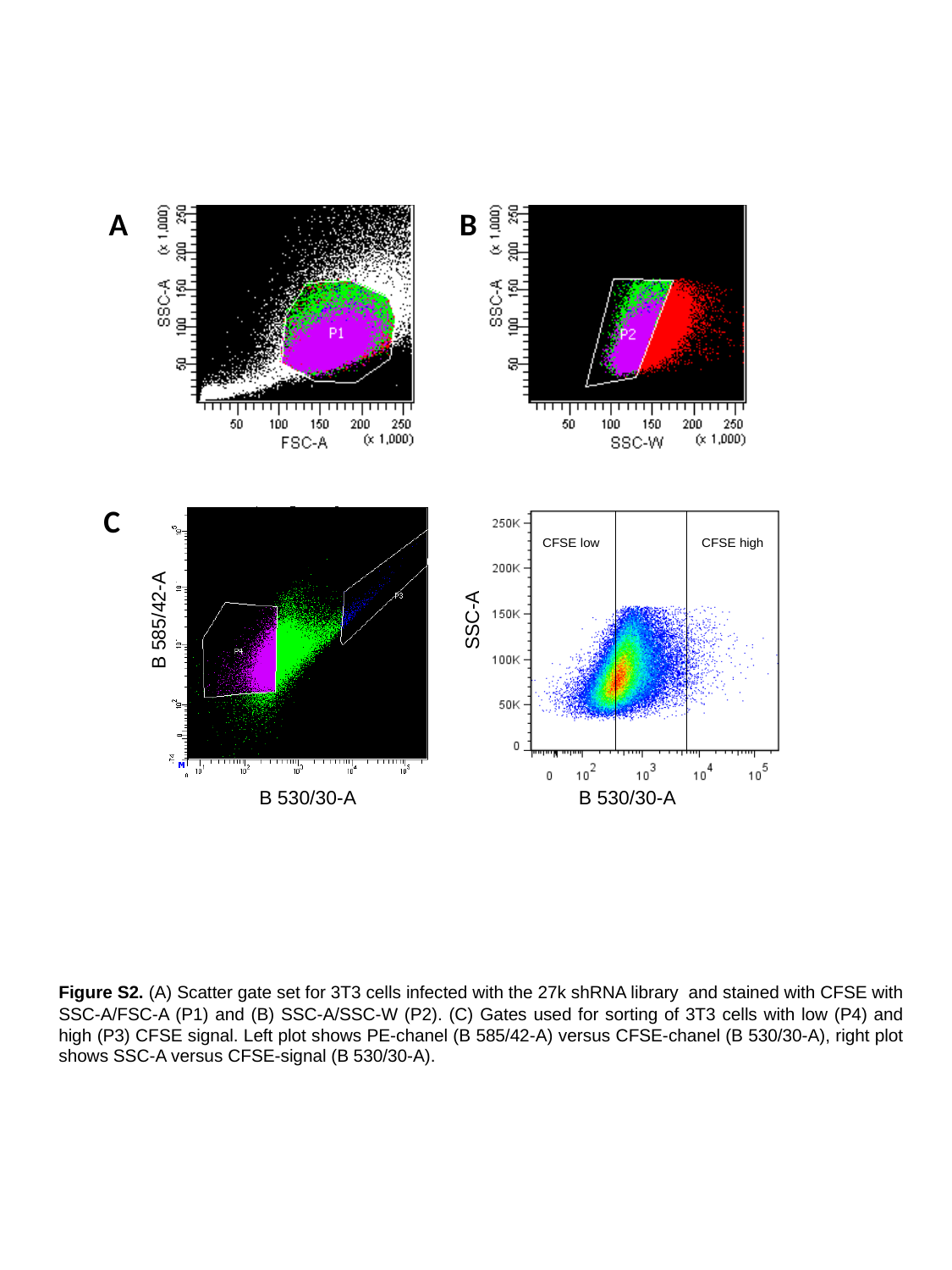

A
B
C
B 585/42-A
SSC-A
B 530/30-A
B 530/30-A
Figure S2. (A) Scatter gate set for 3T3 cells infected with the 27k shRNA library and stained with CFSE with SSC-A/FSC-A (P1) and (B) SSC-A/SSC-W (P2). (C) Gates used for sorting of 3T3 cells with low (P4) and high (P3) CFSE signal. Left plot shows PE-chanel (B 585/42-A) versus CFSE-chanel (B 530/30-A), right plot shows SSC-A versus CFSE-signal (B 530/30-A).

## Slide 4
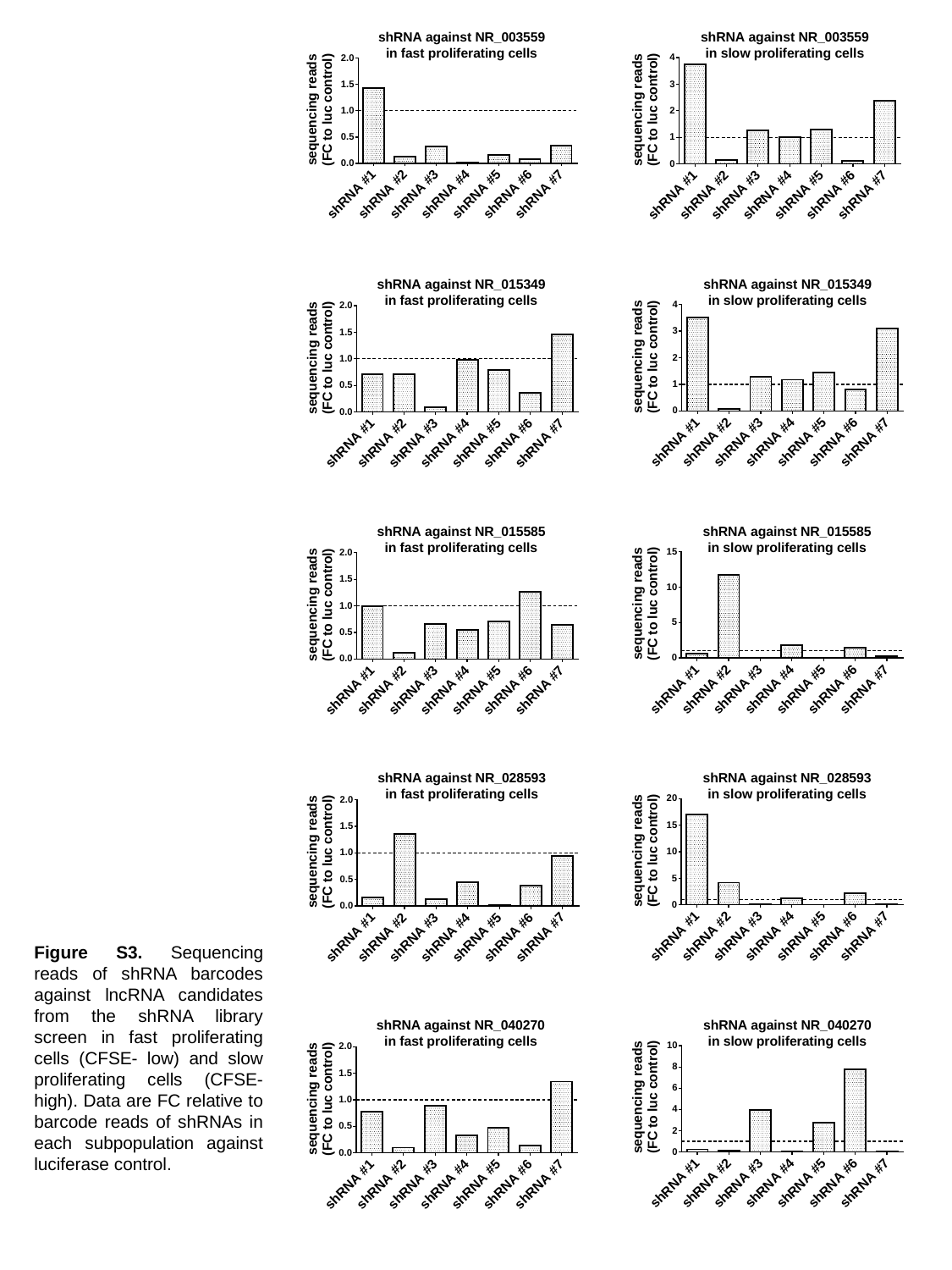

Figure S3. Sequencing reads of shRNA barcodes against lncRNA candidates from the shRNA library screen in fast proliferating cells (CFSE- low) and slow proliferating cells (CFSE-high). Data are FC relative to barcode reads of shRNAs in each subpopulation against luciferase control.

## Slide 5
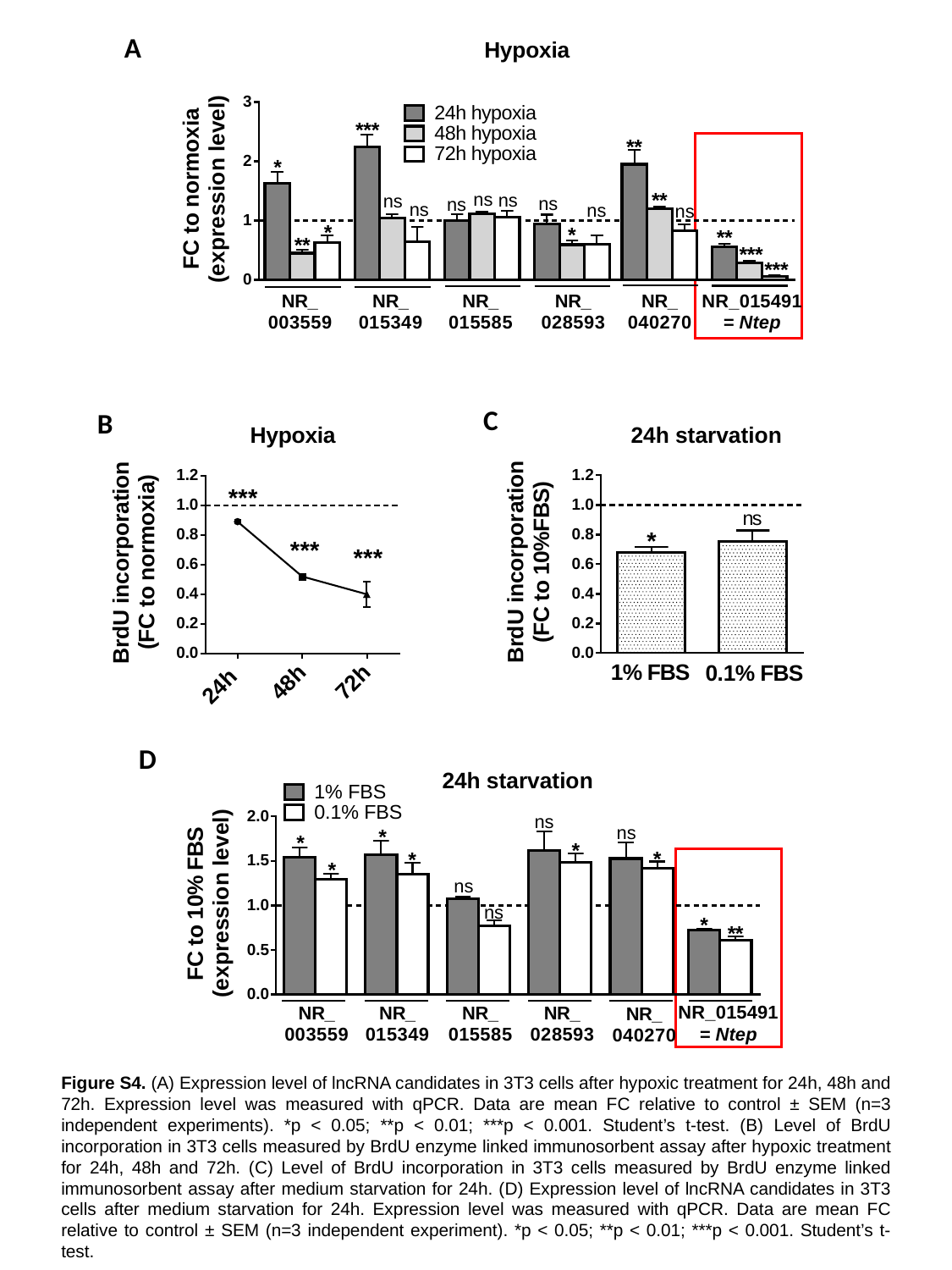

A
C
B
D
Figure S4. (A) Expression level of lncRNA candidates in 3T3 cells after hypoxic treatment for 24h, 48h and 72h. Expression level was measured with qPCR. Data are mean FC relative to control ± SEM (n=3 independent experiments). *p < 0.05; **p < 0.01; ***p < 0.001. Student’s t-test. (B) Level of BrdU incorporation in 3T3 cells measured by BrdU enzyme linked immunosorbent assay after hypoxic treatment for 24h, 48h and 72h. (C) Level of BrdU incorporation in 3T3 cells measured by BrdU enzyme linked immunosorbent assay after medium starvation for 24h. (D) Expression level of lncRNA candidates in 3T3 cells after medium starvation for 24h. Expression level was measured with qPCR. Data are mean FC relative to control ± SEM (n=3 independent experiment). *p < 0.05; **p < 0.01; ***p < 0.001. Student’s t-test.

## Slide 6
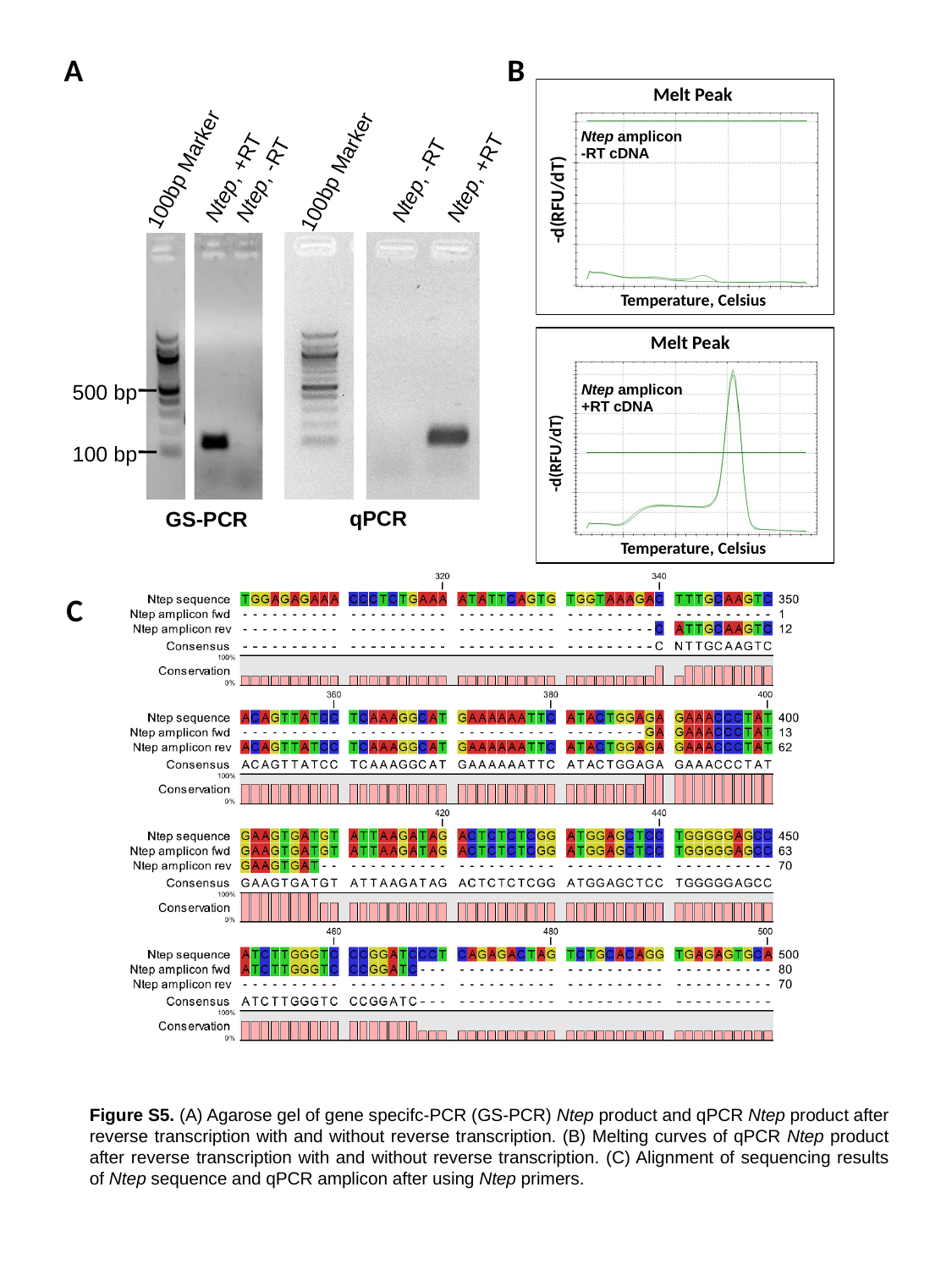

A
B
Ntep, +RT
100bp Marker
Ntep, -RT
qPCR
100bp Marker
Ntep, +RT
Ntep, -RT
GS-PCR
500 bp
100 bp
C
Figure S5. (A) Agarose gel of gene specifc-PCR (GS-PCR) Ntep product and qPCR Ntep product after reverse transcription with and without reverse transcription. (B) Melting curves of qPCR Ntep product after reverse transcription with and without reverse transcription. (C) Alignment of sequencing results of Ntep sequence and qPCR amplicon after using Ntep primers.

## Slide 7
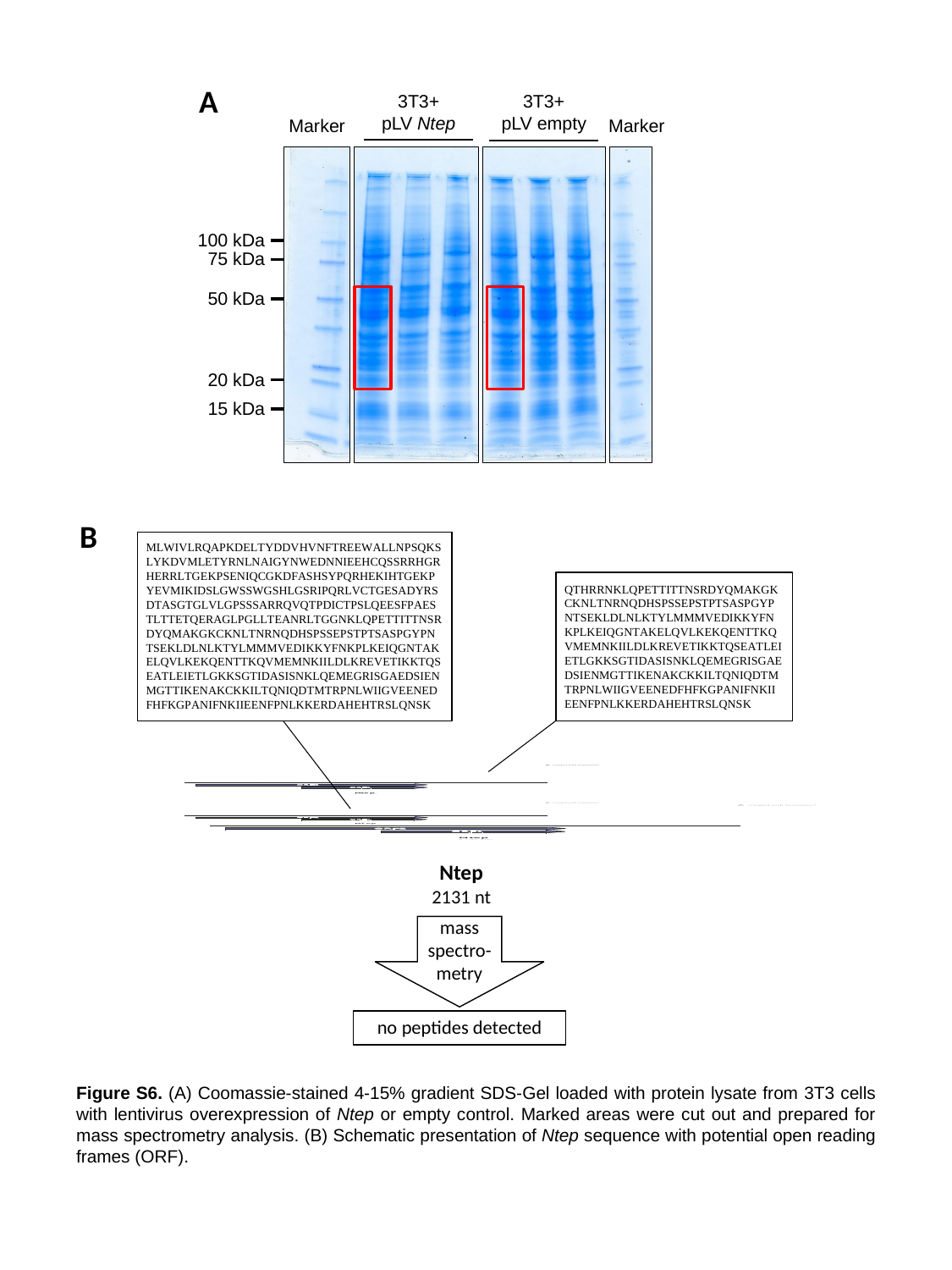

A
B
Figure S6. (A) Coomassie-stained 4-15% gradient SDS-Gel loaded with protein lysate from 3T3 cells with lentivirus overexpression of Ntep or empty control. Marked areas were cut out and prepared for mass spectrometry analysis. (B) Schematic presentation of Ntep sequence with potential open reading frames (ORF).

## Slide 8
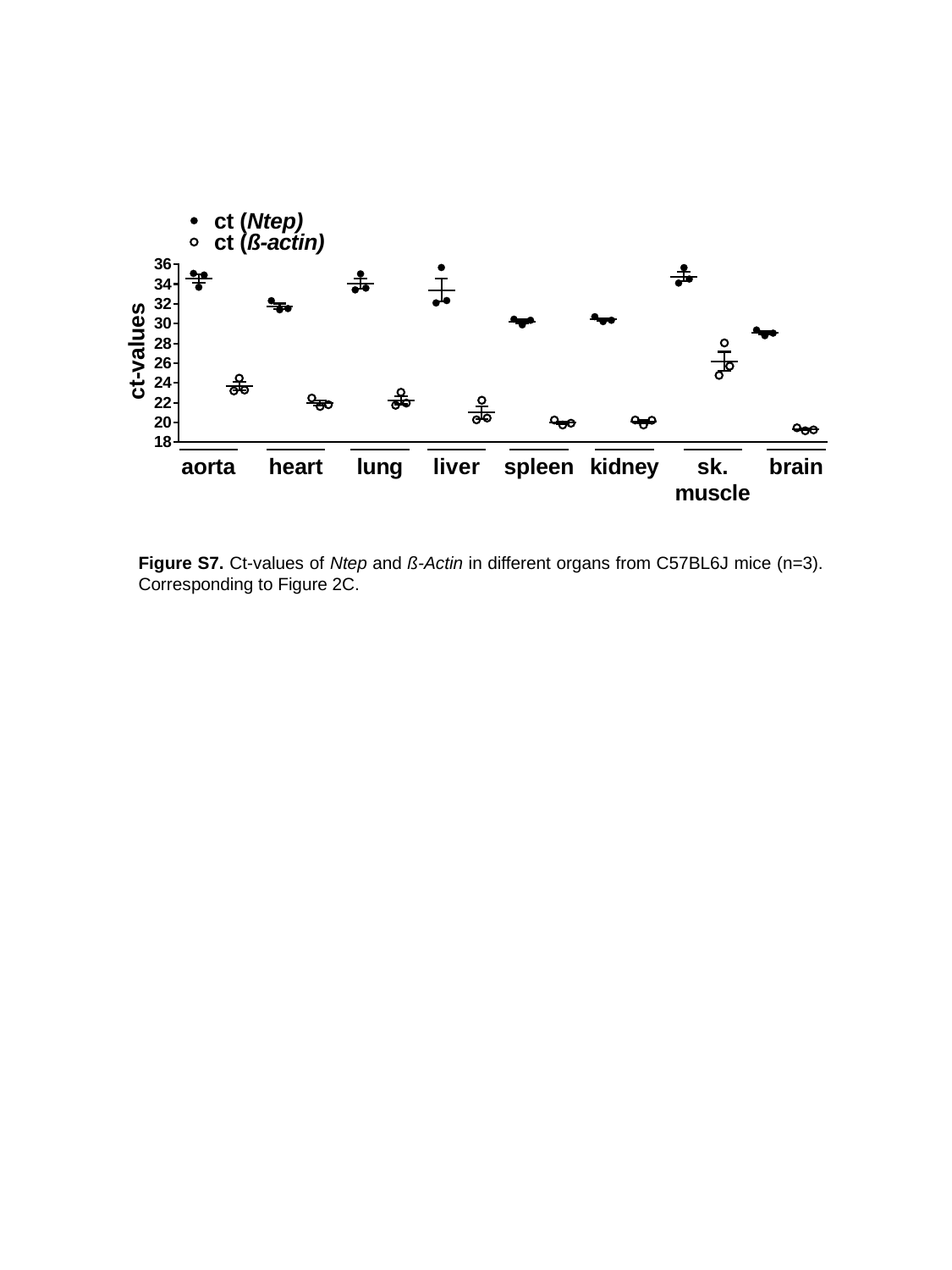

Figure S7. Ct-values of Ntep and ß-Actin in different organs from C57BL6J mice (n=3). Corresponding to Figure 2C.

## Slide 9
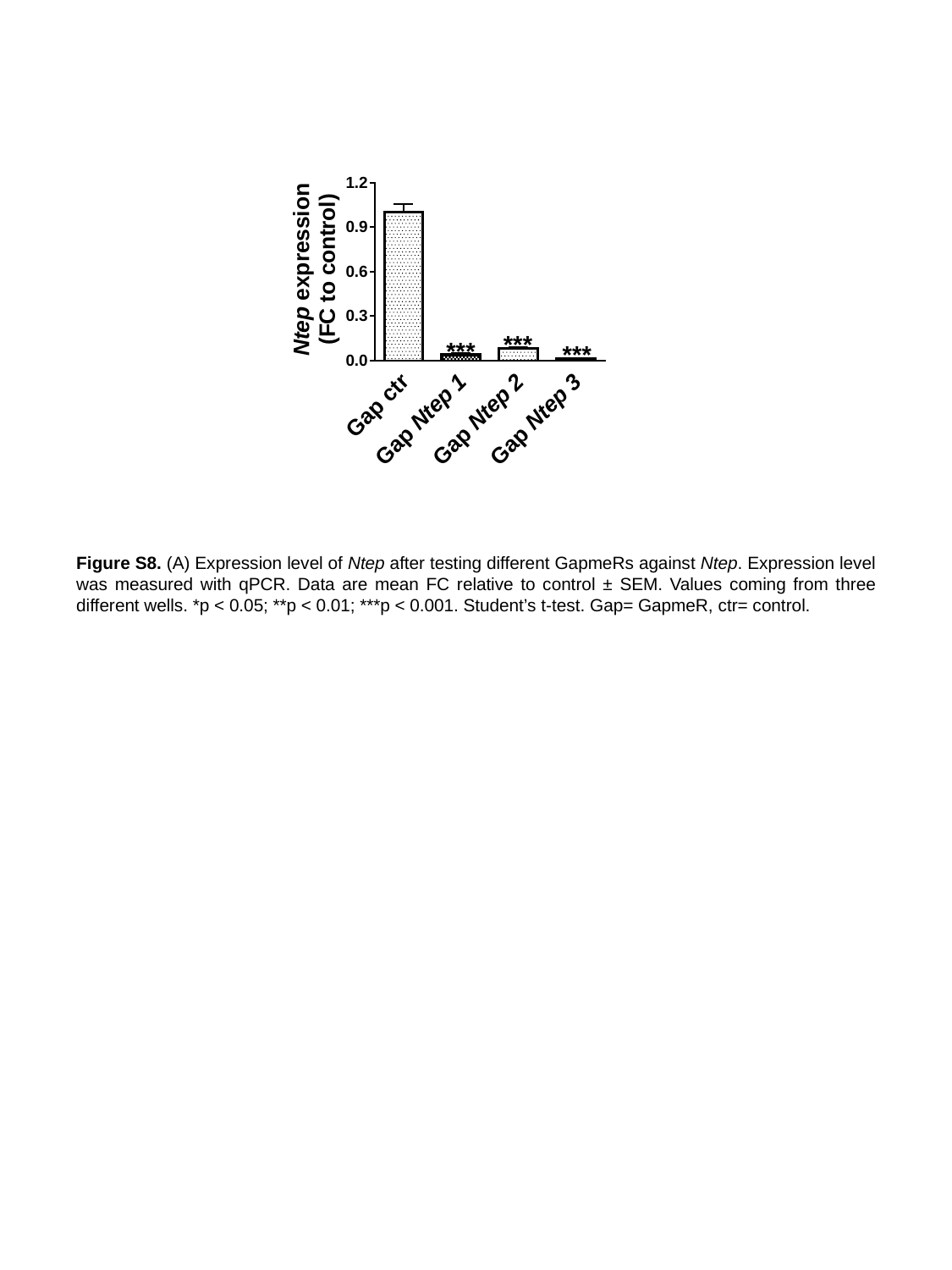

Figure S8. (A) Expression level of Ntep after testing different GapmeRs against Ntep. Expression level was measured with qPCR. Data are mean FC relative to control ± SEM. Values coming from three different wells. *p < 0.05; **p < 0.01; ***p < 0.001. Student’s t-test. Gap= GapmeR, ctr= control.

## Slide 10
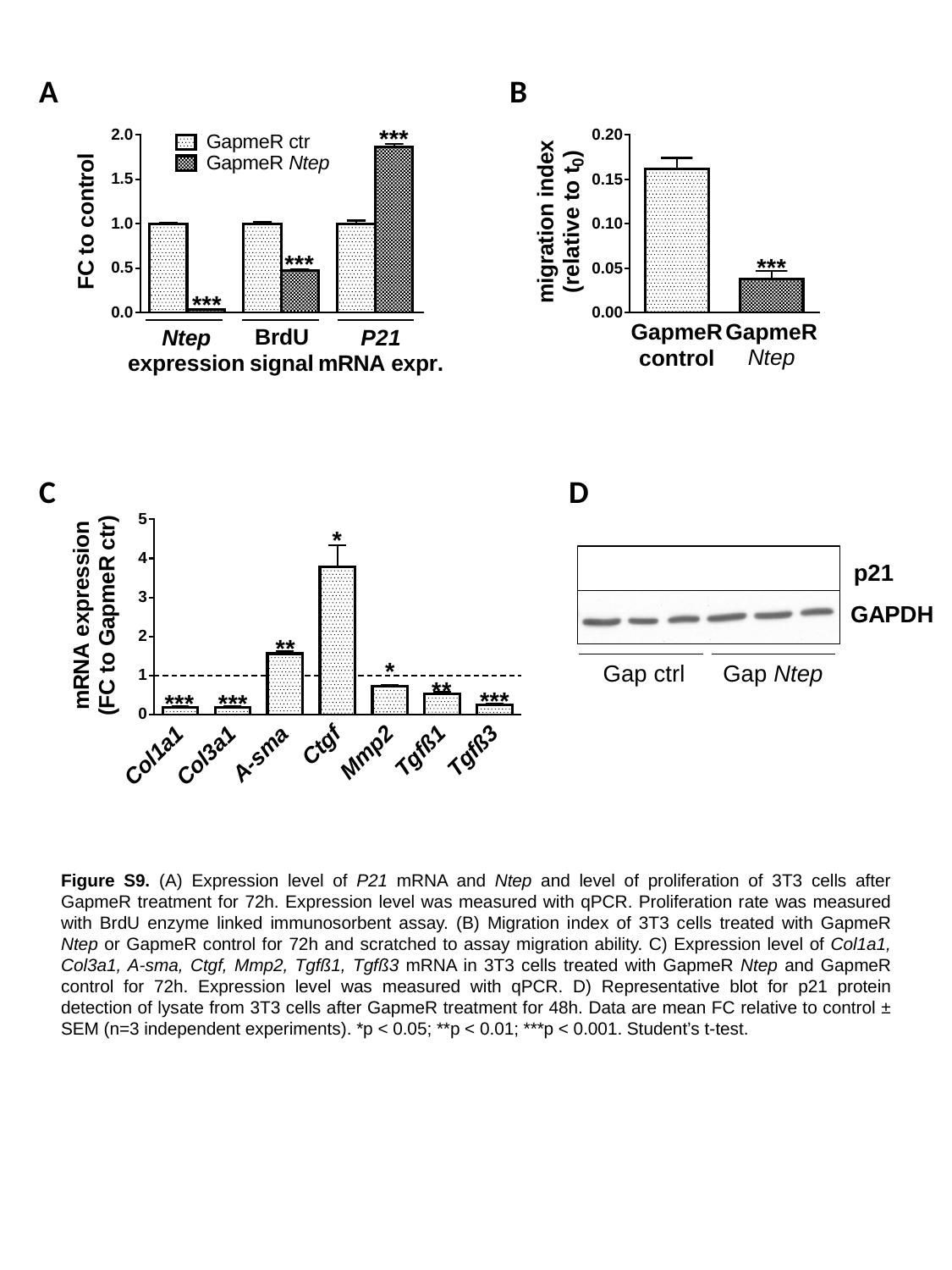

A
B
C
D
Figure S9. (A) Expression level of P21 mRNA and Ntep and level of proliferation of 3T3 cells after GapmeR treatment for 72h. Expression level was measured with qPCR. Proliferation rate was measured with BrdU enzyme linked immunosorbent assay. (B) Migration index of 3T3 cells treated with GapmeR Ntep or GapmeR control for 72h and scratched to assay migration ability. C) Expression level of Col1a1, Col3a1, A-sma, Ctgf, Mmp2, Tgfß1, Tgfß3 mRNA in 3T3 cells treated with GapmeR Ntep and GapmeR control for 72h. Expression level was measured with qPCR. D) Representative blot for p21 protein detection of lysate from 3T3 cells after GapmeR treatment for 48h. Data are mean FC relative to control ± SEM (n=3 independent experiments). *p < 0.05; **p < 0.01; ***p < 0.001. Student’s t-test.

## Slide 11
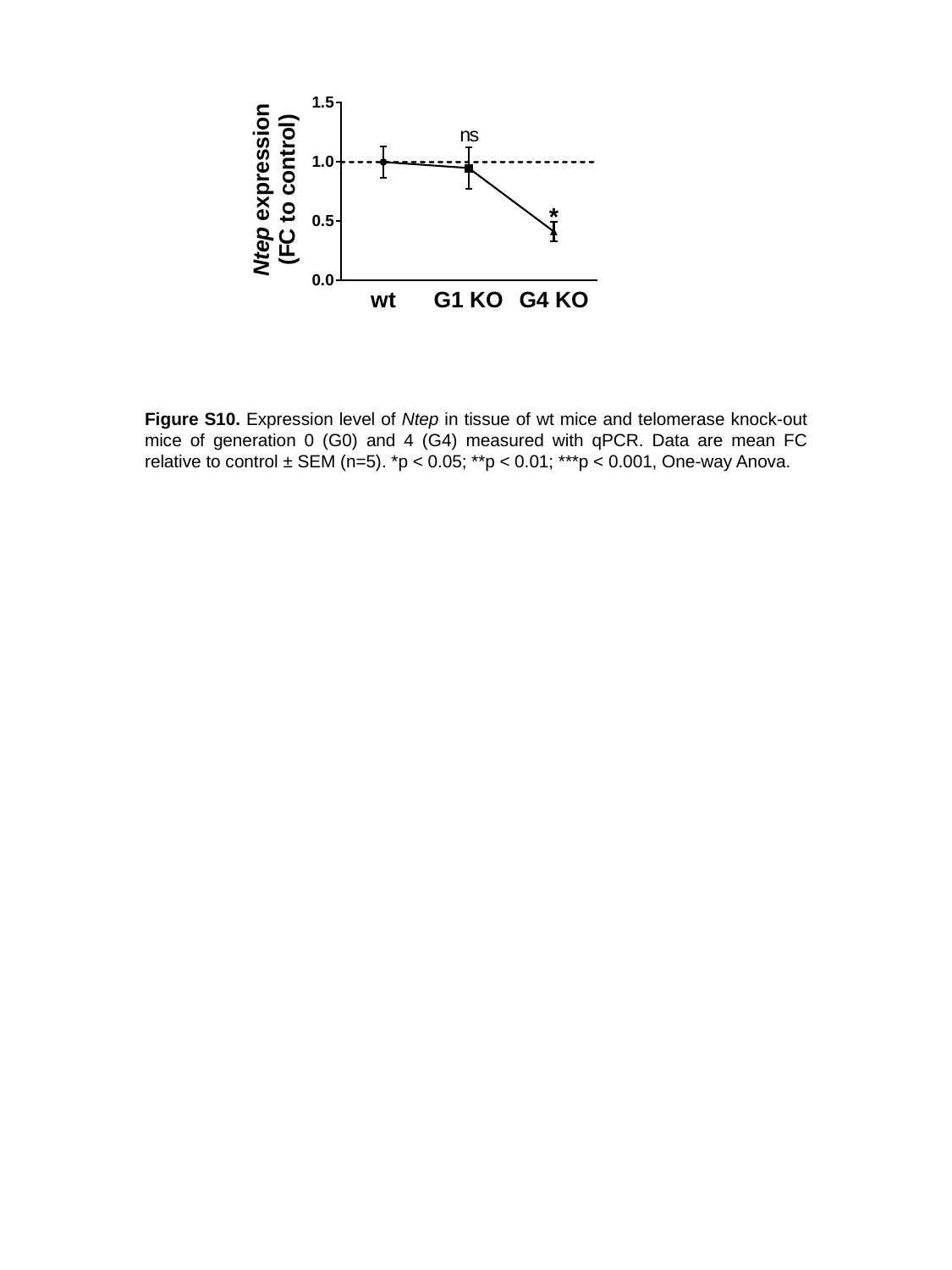

Figure S10. Expression level of Ntep in tissue of wt mice and telomerase knock-out mice of generation 0 (G0) and 4 (G4) measured with qPCR. Data are mean FC relative to control ± SEM (n=5). *p < 0.05; **p < 0.01; ***p < 0.001, One-way Anova.

## Slide 12
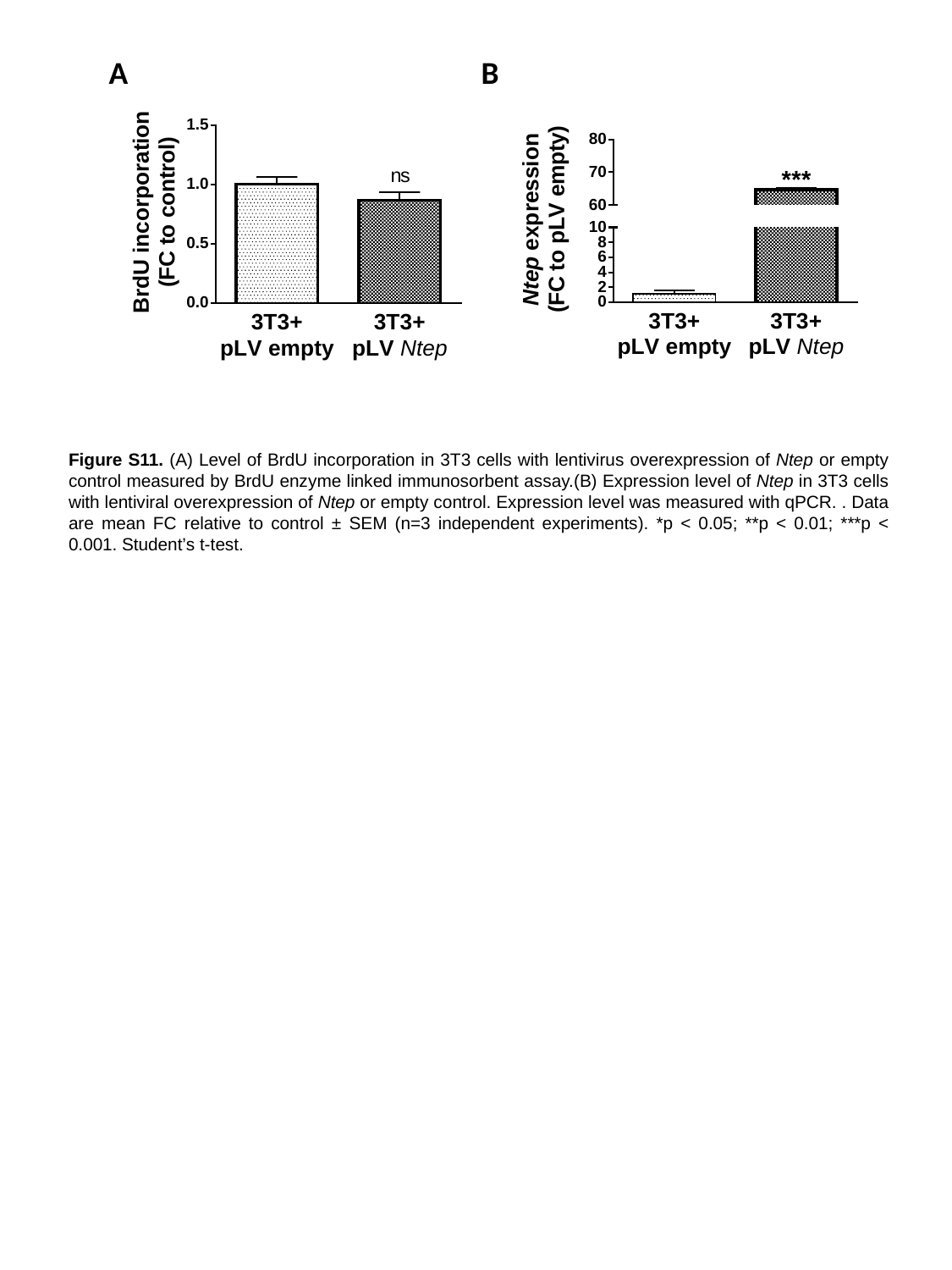

A
B
Figure S11. (A) Level of BrdU incorporation in 3T3 cells with lentivirus overexpression of Ntep or empty control measured by BrdU enzyme linked immunosorbent assay.(B) Expression level of Ntep in 3T3 cells with lentiviral overexpression of Ntep or empty control. Expression level was measured with qPCR. . Data are mean FC relative to control ± SEM (n=3 independent experiments). *p < 0.05; **p < 0.01; ***p < 0.001. Student’s t-test.

## Slide 13
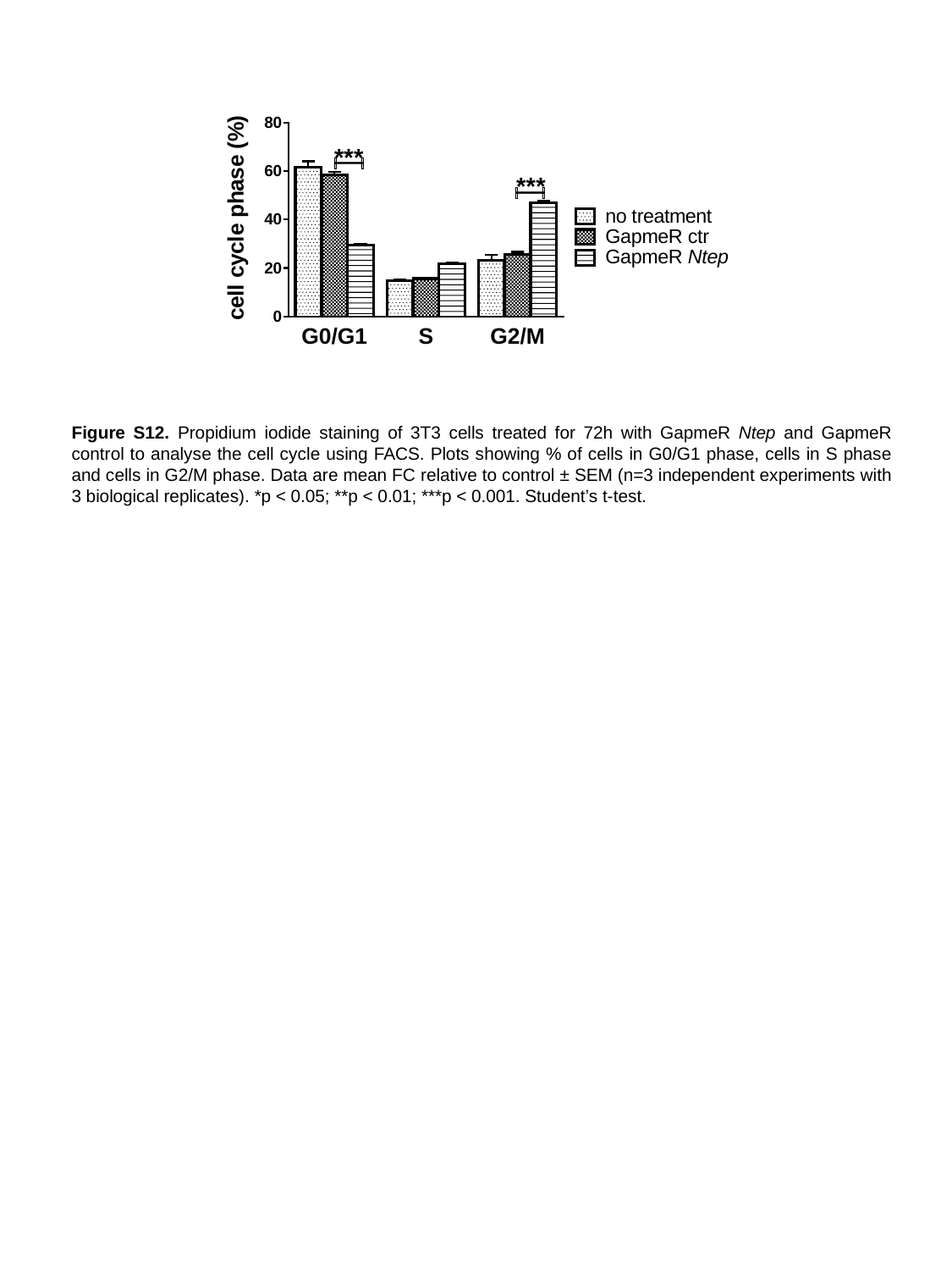

Figure S12. Propidium iodide staining of 3T3 cells treated for 72h with GapmeR Ntep and GapmeR control to analyse the cell cycle using FACS. Plots showing % of cells in G0/G1 phase, cells in S phase and cells in G2/M phase. Data are mean FC relative to control ± SEM (n=3 independent experiments with 3 biological replicates). *p < 0.05; **p < 0.01; ***p < 0.001. Student’s t-test.

## Slide 14
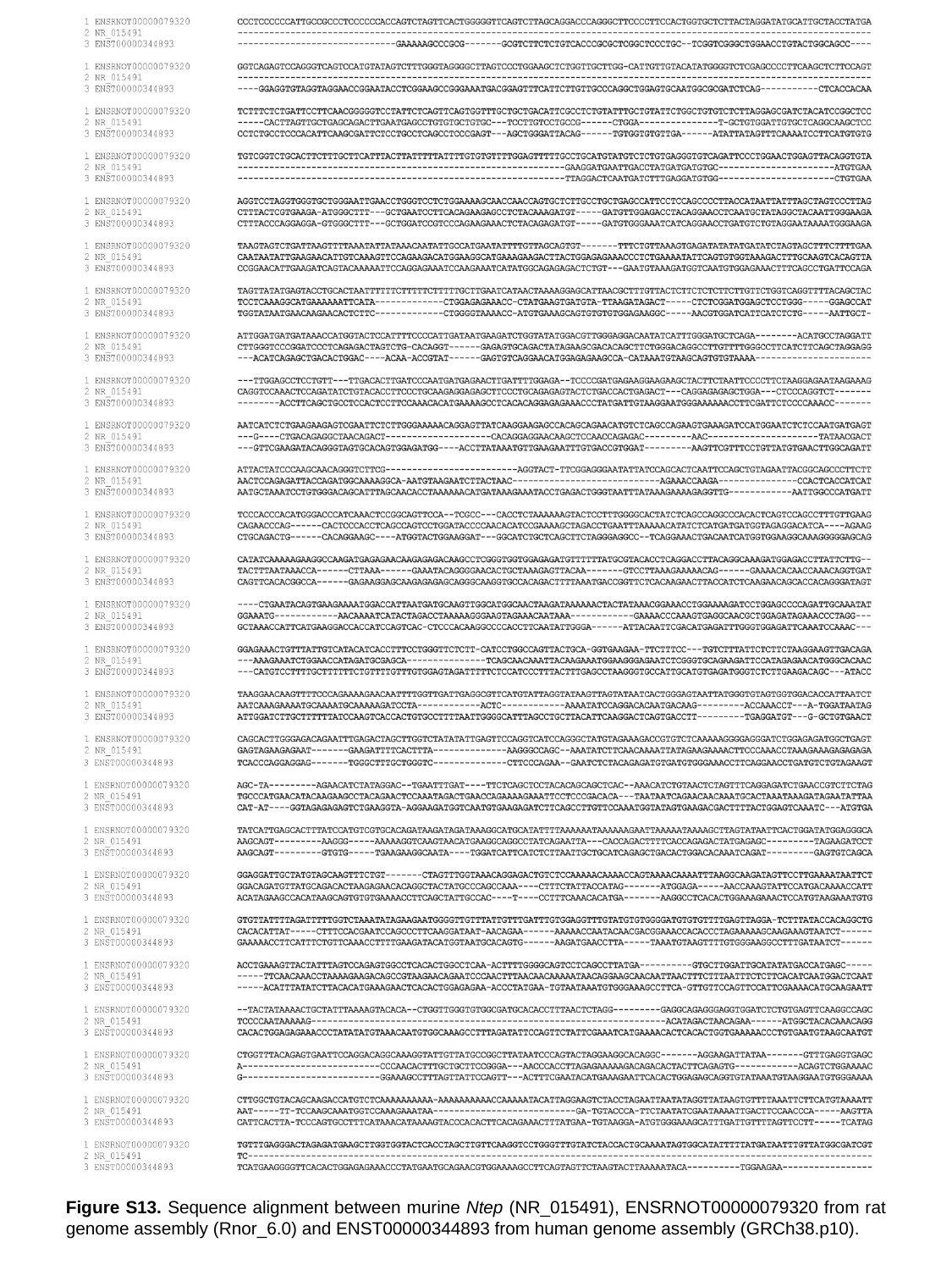

Figure S13. Sequence alignment between murine Ntep (NR_015491), ENSRNOT00000079320 from rat genome assembly (Rnor_6.0) and ENST00000344893 from human genome assembly (GRCh38.p10).

## Slide 15
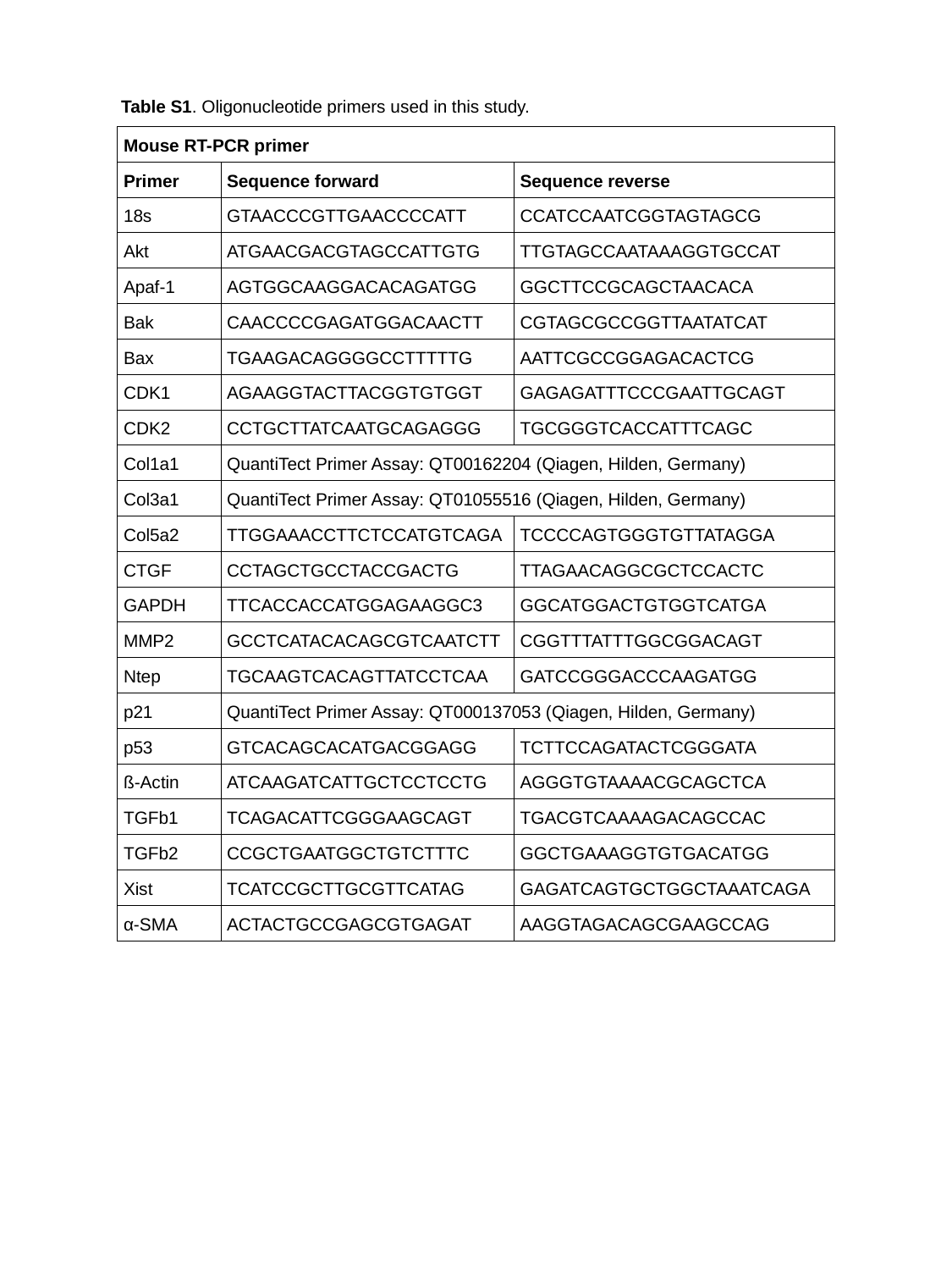

Table S1. Oligonucleotide primers used in this study.
| Mouse RT-PCR primer | | |
| --- | --- | --- |
| Primer | Sequence forward | Sequence reverse |
| 18s | GTAACCCGTTGAACCCCATT | CCATCCAATCGGTAGTAGCG |
| Akt | ATGAACGACGTAGCCATTGTG | TTGTAGCCAATAAAGGTGCCAT |
| Apaf-1 | AGTGGCAAGGACACAGATGG | GGCTTCCGCAGCTAACACA |
| Bak | CAACCCCGAGATGGACAACTT | CGTAGCGCCGGTTAATATCAT |
| Bax | TGAAGACAGGGGCCTTTTTG | AATTCGCCGGAGACACTCG |
| CDK1 | AGAAGGTACTTACGGTGTGGT | GAGAGATTTCCCGAATTGCAGT |
| CDK2 | CCTGCTTATCAATGCAGAGGG | TGCGGGTCACCATTTCAGC |
| Col1a1 | QuantiTect Primer Assay: QT00162204 (Qiagen, Hilden, Germany) | |
| Col3a1 | QuantiTect Primer Assay: QT01055516 (Qiagen, Hilden, Germany) | |
| Col5a2 | TTGGAAACCTTCTCCATGTCAGA | TCCCCAGTGGGTGTTATAGGA |
| CTGF | CCTAGCTGCCTACCGACTG | TTAGAACAGGCGCTCCACTC |
| GAPDH | TTCACCACCATGGAGAAGGC3 | GGCATGGACTGTGGTCATGA |
| MMP2 | GCCTCATACACAGCGTCAATCTT | CGGTTTATTTGGCGGACAGT |
| Ntep | TGCAAGTCACAGTTATCCTCAA | GATCCGGGACCCAAGATGG |
| p21 | QuantiTect Primer Assay: QT000137053 (Qiagen, Hilden, Germany) | |
| p53 | GTCACAGCACATGACGGAGG | TCTTCCAGATACTCGGGATA |
| ß-Actin | ATCAAGATCATTGCTCCTCCTG | AGGGTGTAAAACGCAGCTCA |
| TGFb1 | TCAGACATTCGGGAAGCAGT | TGACGTCAAAAGACAGCCAC |
| TGFb2 | CCGCTGAATGGCTGTCTTTC | GGCTGAAAGGTGTGACATGG |
| Xist | TCATCCGCTTGCGTTCATAG | GAGATCAGTGCTGGCTAAATCAGA |
| α-SMA | ACTACTGCCGAGCGTGAGAT | AAGGTAGACAGCGAAGCCAG |

## Slide 16
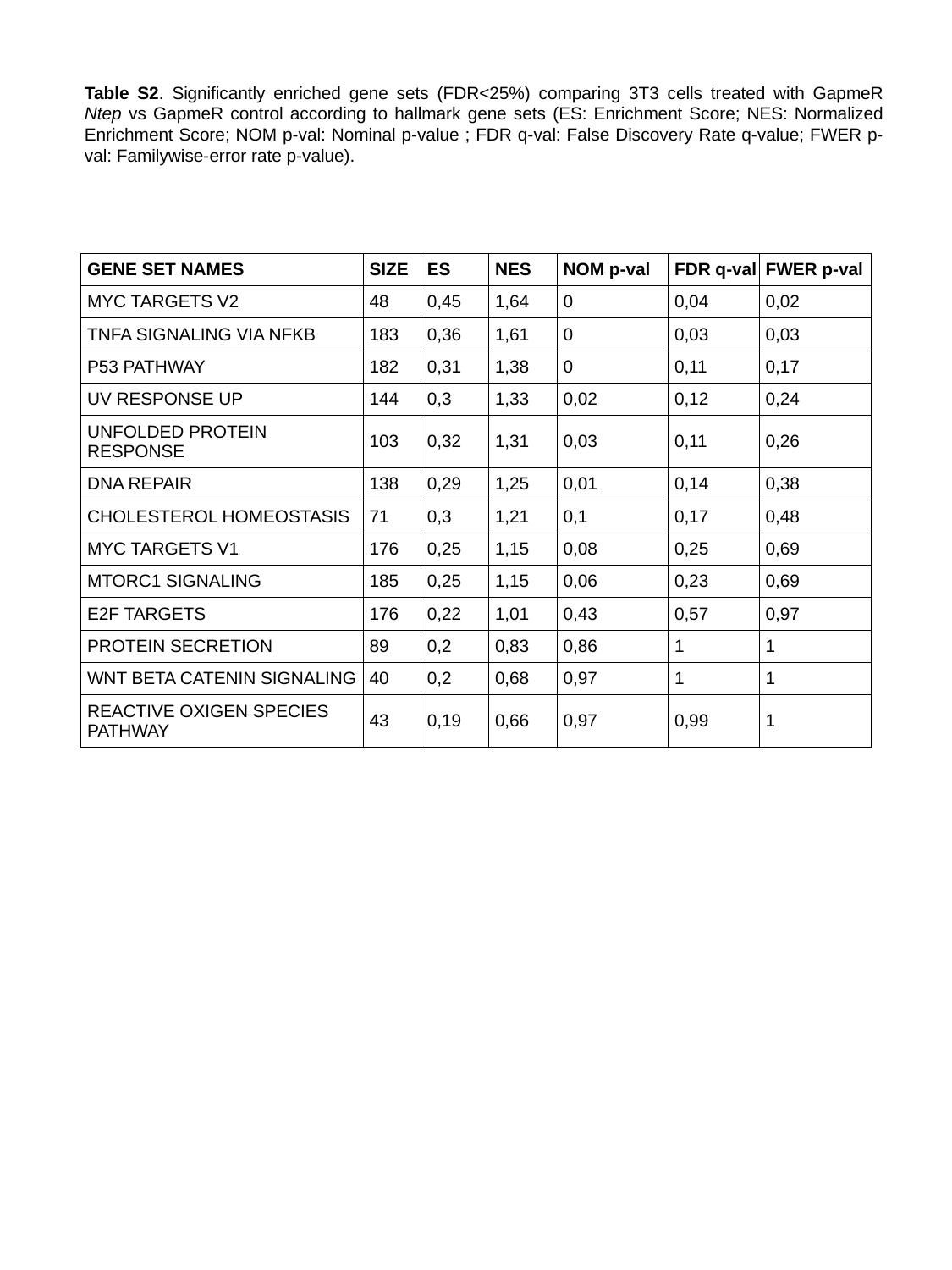

Table S2. Significantly enriched gene sets (FDR<25%) comparing 3T3 cells treated with GapmeR Ntep vs GapmeR control according to hallmark gene sets (ES: Enrichment Score; NES: Normalized Enrichment Score; NOM p-val: Nominal p-value ; FDR q-val: False Discovery Rate q-value; FWER p-val: Familywise-error rate p-value).
| GENE SET NAMES | SIZE | ES | NES | NOM p-val | FDR q-val | FWER p-val |
| --- | --- | --- | --- | --- | --- | --- |
| MYC TARGETS V2 | 48 | 0,45 | 1,64 | 0 | 0,04 | 0,02 |
| TNFA SIGNALING VIA NFKB | 183 | 0,36 | 1,61 | 0 | 0,03 | 0,03 |
| P53 PATHWAY | 182 | 0,31 | 1,38 | 0 | 0,11 | 0,17 |
| UV RESPONSE UP | 144 | 0,3 | 1,33 | 0,02 | 0,12 | 0,24 |
| UNFOLDED PROTEIN RESPONSE | 103 | 0,32 | 1,31 | 0,03 | 0,11 | 0,26 |
| DNA REPAIR | 138 | 0,29 | 1,25 | 0,01 | 0,14 | 0,38 |
| CHOLESTEROL HOMEOSTASIS | 71 | 0,3 | 1,21 | 0,1 | 0,17 | 0,48 |
| MYC TARGETS V1 | 176 | 0,25 | 1,15 | 0,08 | 0,25 | 0,69 |
| MTORC1 SIGNALING | 185 | 0,25 | 1,15 | 0,06 | 0,23 | 0,69 |
| E2F TARGETS | 176 | 0,22 | 1,01 | 0,43 | 0,57 | 0,97 |
| PROTEIN SECRETION | 89 | 0,2 | 0,83 | 0,86 | 1 | 1 |
| WNT BETA CATENIN SIGNALING | 40 | 0,2 | 0,68 | 0,97 | 1 | 1 |
| REACTIVE OXIGEN SPECIES PATHWAY | 43 | 0,19 | 0,66 | 0,97 | 0,99 | 1 |
